# A unique insight into the MiRNA profile during genital chlamydial infection

**DOI:** 10.1186/s12864-019-5495-6

**Published:** 2019-02-18

**Authors:** Ifeyinwa Benyeogor, Tankya Simoneaux, Yuehao Wu, Stephanie Lundy, Zenas George, Khamia Ryans, Danielle McKeithen, Roshan Pais, Debra Ellerson, W. Walter Lorenz, Tolulope Omosun, Winston Thompson, Francis O. Eko, Carolyn M. Black, Uriel Blas-Machado, Joseph U. Igietseme, Qing He, Yusuf Omosun

**Affiliations:** 1 Department of Microbiology, Biochemistry & Immunology, Morehouse School of Medicine, 720 Westview Drive, S.W, Atlanta, GA 30310 USA; 2 Centers for Disease Control & Prevention (CDC), Atlanta, GA 30333 USA; 3 Department of Pathology, University of Georgia, College of Veterinary Medicine, Athens, GA 30602 USA; 4 Institute of Bioinformatics, University of Georgia, Athens, GA 30602 USA; 5 Department of Physical Sciences, Georgia State University, Covington, GA 30014 USA; 6 Department of Physiology, Morehouse School of Medicine, Atlanta, GA 30310 USA

**Keywords:** miRNAs, Differential expression, Chlamydia infection, TFI, Chlamydial pathogenesis

## Abstract

**Background:**

Genital *C. trachomatis* infection may cause pelvic inflammatory disease (PID) that can lead to tubal factor infertility (TFI). Understanding the pathogenesis of chlamydial complications including the pathophysiological processes within the female host genital tract is important in preventing adverse pathology. MicroRNAs regulate several pathophysiological processes of infectious and non-infectious etiologies. In this study, we tested the hypothesis that the miRNA profile of single and repeat genital chlamydial infections will be different and that these differences will be time dependent. Thus, we analyzed and compared differentially expressed mice genital tract miRNAs after single and repeat chlamydia infections using a *C. muridarum* mouse model. Mice were sacrificed and their genital tract tissues were collected at 1, 2, 4, and 8 weeks after a single and repeat chlamydia infections. Histopathology, and miRNA sequencing were performed.

**Results:**

Histopathology presentation showed that the oviduct and uterus of reinfected mice were more inflamed, distended and dilated compared to mice infected once. The miRNAs expression profile was different in the reproductive tissues after a reinfection, with a greater number of miRNAs expressed after reinfection. Also, the number of miRNAs expressed each week after chlamydia infection and reinfection varied, with weeks eight and one having the highest number of differentially expressed miRNAs for chlamydia infection and reinfection respectively. Ten miRNAs; mmu-miR-378b, mmu-miR-204-5p, mmu-miR-151-5p, mmu-miR-142-3p, mmu-miR-128-3p, mmu-miR-335-3p, mmu-miR-195a-3p, mmu-miR-142-5p, mmu-miR-106a-5p and mmu-miR-92a-3p were common in both primary chlamydia infection and reinfection. Pathway analysis showed that, amongst other functions, the differentially regulated miRNAs control pathways involved in cellular and tissue development, disease conditions and toxicity.

**Conclusions:**

This study provides insights into the changes in miRNA expression over time after chlamydia infection and reinfection, as well as the pathways they regulate to determine pathological outcomes. The miRNAs networks generated in our study shows that there are differences in the focus molecules involved in significant biological functions in chlamydia infection and reinfection, implying that chlamydial pathogenesis occurs differently for each type of infection and that this could be important when determining treatments regime and disease outcome. The study underscores the crucial role of host factors in chlamydia pathogenesis.

**Electronic supplementary material:**

The online version of this article (10.1186/s12864-019-5495-6) contains supplementary material, which is available to authorized users.

## Background

Chlamydia infection is one of the most common bacterial sexually transmitted infection worldwide. The complications in women include endometritis, salpingitis, ectopic pregnancy, pelvic inflammatory disease (PID) and tubal factor infertility (TFI) [1, 2]. Approximately one-third of PID attributed to *Chlamydia trachomatis* infection occurs mainly in reproductive-age women [3]. Several clinical syndromes are associated with PID caused by *Chlamydia*, which during physical examinations presents as pelvic or lower abdominal pain or uterine or adnexal tenderness [4]. Increased levels of inflammatory markers such as mucin 16, erythrocyte sedimentation rate, C-reactive protein, incidence of tubo-ovarian abscess, ectopic pregnancy, tubal infertility, Fitz-Hugh-Curtis syndrome and longer hospitalization are also associated with genital chlamydia infection in acute PID [5].

The developmental cycle of *C. trachomatis* alternates between the extracellular infectious elementary body and the intracellular, non-infectious reticulate body [6]. Columnar and transitional epithelial cells are the primary cells infected by *Chlamydia* [7]. *C. trachomatis* being an intracellular obligate parasite employs an array of host processes to support its developmental cycle [8]. This manipulation of host processes increases influx of innate cells, release of tissue damaging proteins and pro-inflammatory cytokines [8, 9]. The intense and chronic inflammatory response is maintained by reinfection or persistent infection in host cells with chlamydial infection ultimately leads to PID [2, 7, 8]. We have previously suggested that host inflammatory and antimicrobial immune responses to infection are important in determining *Chlamydia*-induced tubal pathology [10, 11]; therefore, balancing these two factors is important in controlling genital chlamydial infections and preventing the sequelae. The initiation of the pathologic process of epithelial-mesenchyme transition in host epithelial cells has been shown to be important during chlamydial infection [12, 13]. This process may progress to fibrosis and fertility-related epithelial dysfunction and provides the co-factor function for herpes virus (HPV)-related cervical epithelial carcinoma [13].

Proposed mechanisms for the inflammatory response and sequalae in Chlamydia infected host cells includes presence of chlamydia proteins and small non-coding RNAs (miRNAs) belonging to the host expressed during *Chlamydia trachomatis* infection [10, 13–18]. miRNAs are evolutionarily conserved, endogenous non-coding RNAs of approximately 22 nucleotides that play important regulatory roles in animals and plants by targeting mRNAs for degradation, cleavage, translational repression or occasional enhancement [19, 20]. miRNAs have been reported to account for approximately one-third of mammalian gene expression by regulating the expression of genes involved in cellular differentiation, maintenance of cellular integrity, development, functions and normal metabolism, reproduction, fibrosis and oncogenesis [21]. miRNAs have been shown to be differentially expressed during ocular chlamydia infection [22, 23]; in addition, we and other authors have shown an association between genital chlamydia infection and differential expression of miRNAs [10]. Furthermore, we showed a direct relationship between the integrity of Dicer, a ribonuclease III enzyme, and key micro-RNAs in the reproductive system [10]. Immune cell type-specific miRNAs appear to regulate the effective immune response to chlamydial infection [24]. The development of PID due to chlamydial infection with virulent and attenuated phenotypes can be predicted using miRNAs [17] with the *Chlamydia* virulence associated with the miRNA expression profile of the host [25]. miRNAs have also been associated with regulating mitochondrial function which is necessary for *C. trachomatis* development [26]. These studies buttress the importance of miRNAs as important factors in host cell changes and response during chlamydial infection.

Although these findings show the importance of miRNAs expression in determining the outcome of chlamydial infections, they however, do not provide a full picture of the role miRNAs may play during chlamydial infection. For instance, they do not show the effect of *Chlamydia trachomatis* on miRNA expression over an extended period. The methods used to analyze genital tract miRNAs so far have not provided a detailed enumeration of all the known miRNAs associated with chlamydial infection. To address this knowledge gap, we investigated the total miRNA expression profile using deep sequencing, in mice infected and reinfected with *C. muridarum*. *C. muridarum* mouse models results in genital tract infections that are similar to acute genital tract *C. trachomatis* infections in women. The infection in mice can cause hydrosalphinx, fibrosis and infertility, which are common in infected women [27–29]. Here, we report the expression profile of miRNAs in the female mouse genital tracts at one, two, four and 8 weeks after chlamydia infection and reinfection.

## Results

### Mice reinfected with *C. muridarum* have pronounced uterine pathology

**Fig. 1:**
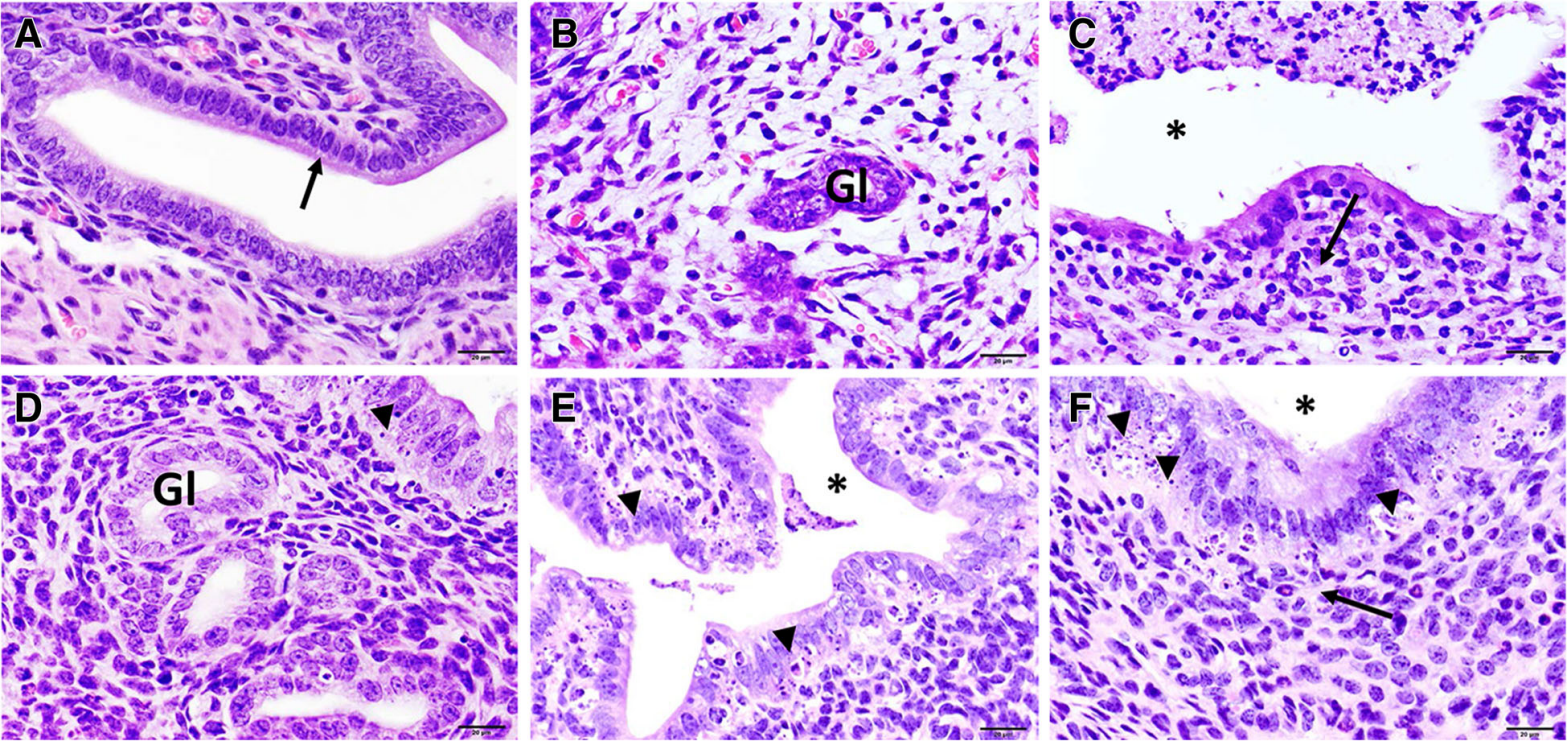
Histopathological assessment of genital tract of mice infected once with *Chlamydia.* **a**. Representative image of the endometrium from an uninfected mouse. A single layer of columnar epithelia (arrow), with basally located nuclei lining the endometrium. The underlying lamina propria has scattered spindled cells, some capillaries, and occasional leukocytes. Hematoxylin and eosin (HE) stain. Scale bar = 20 μm. **b** and **c**. Representative image of uterine endometrium with increased presence of eosinophils after the first and second weeks of infection. The lamina propria is mildly hypercellular, with loosely arranged spindled stromal cells and increased numbers of eosinophils around an endometrial gland (Gl). HE stain. Scale bar 20 μm. **d**-**f**. Representative images of inflammed uterine endometrium with necrosis after 4 weeks of infection. **d**. The uterine lumen (asterisks) is filled with necrotic cellular debris. The mucosa has widespread epithelial apoptotic necrosis (arrowheads). The lamina propria is moderately hypercellular, with increased numbers of neutrophils, eosinophils, and few lymphocytes (arrows). HE stain. Scale bar 20 μm

**Fig. 2:**
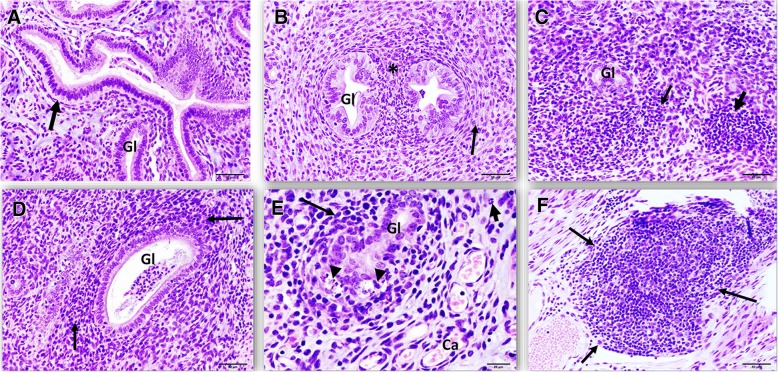
Histopathological assessment of genital tract of mice infected twice with *Chlamydia*. **a**. Representative image of the endometrium from an uninfected mouse. A single layer of columnar epithelia (arrow), with basally located nuclei lining the endometrium. The underlying lamina propria has scattered spindled cells, some capillaries, and occasional leukocytes in a pale basophilic myxomatous matrix. (Endometrial gland = Gl). HE stain. Scale bar 50 μm. **b**. Representative image of uterine endometrium with periglandular fibrosis after 1 week of reinfection. Increased numbers of neutrophils (asterisk) and spindled cells (fibroblasts, arrow) are arranged around endometrial glands (Gl). HE stain. Scale bar 50 μm. **c**. Representative image of uterine endometrium with increased lymphocyte presence after 2 weeks of reinfection. Large numbers of lymphocytes (arrows) obscure and expand the tissues around an endometrial gland (Gl). HE stain. Scale bar 50 μm. **d** and **e**. Representative image of uterine endometrium with increased lymphocyte presence after 4 weeks of reinfection with lymphocytic endometritis. Large numbers of lymphocytes, with fewer plasma cells (long arrows) and occasional neutrophils (short arrow) obscure and expand the tissues around an endometrial gland (Gl). Arrowheads point to foci of glandular apoptotic necrosis. Capillaries = Ca. HE stain. Scale bars 50 and 20 μm, respectively. **f**. Representative image of uterine endometrium with increased lymphocyte presence and lymphoid follicle formation (arrows) after 8 weeks of reinfection. An expansive lymphoid follicle obscures and expands the tissues of the deep stratum spongiosum and adjacent myometrium. HE stain. Scale bar 50 μm

**Fig. 3:**
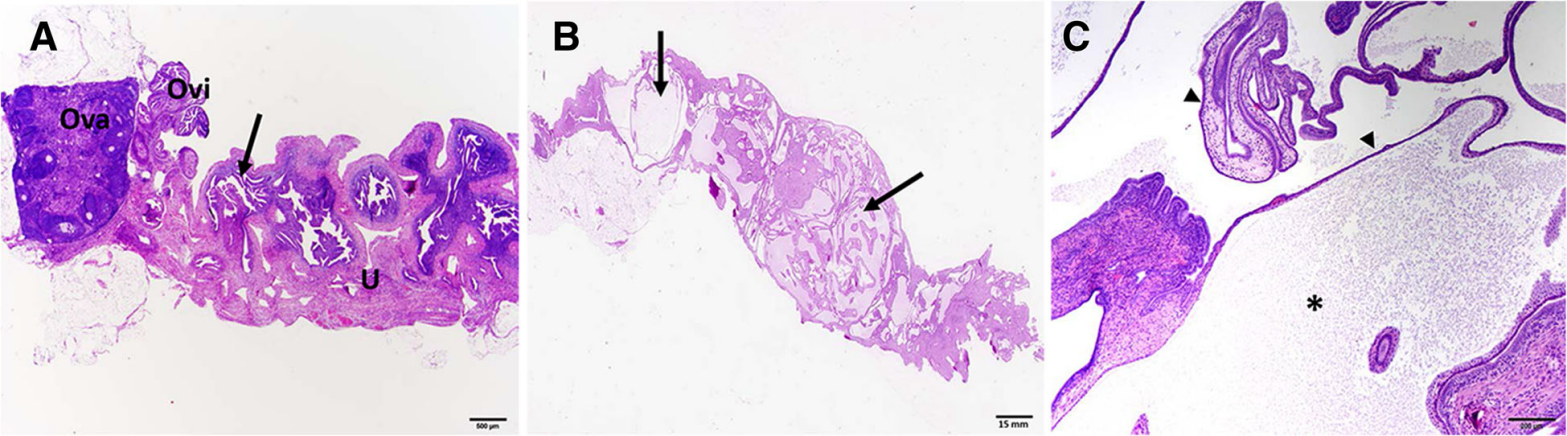
Further Histopathological assessment of genital tract of mice infected twice with *Chlamydia.* **a**. Representative, longitudinal image of an uninfected mouse uterus (U), oviduct (Ovi), and ovary (Ova). A single layer of columnar epithelia (arrow), with basally located nuclei lining the endometrium (arrow). HE stain. Scale bar 500 μm. **b** and **c**. Representative, sub-gross (**b**) and close up (**c**) images of a mouse uterus with marked cystic endometrial hyperplasia (arrows) 8 weeks after reinfection. Branched, papillary projections (arrowheads) of endometrium extend into the cystically dilated uterine lumen (asterisk). HE stain. Scale bars are 15 mm and 200 μm, respectively

We performed histopathological examinations of the genital tract tissues from *C. muridarum* infected and reinfected mice and compared the changes. Significant findings were mainly detected in the uterus. When compared to controls, mice infected once had a number of lesions that increased with time, meaning that more lessions were formed in mice genital tracts as the infection progressed. The pathologic findings varied from eosinophilic changes in the endometrium to lymphocytic inflammation, with associated apoptotic necrosis of endometrial epithelia (Fig. 1a-f). Reinfected mice had an increased number of tissue alterations in the uterus and oviduct, compared to control mice. The number of lesions was not associated with time (Fig. 2a-f), but included periglandular inflammation with and without the formation of new fibrous tissue (periglandular fibroplasia) (Fig. 2b and c). The character of the periglandular inflammation was predominantly lymphocytic and plasmacytic, with formation of lymphoid follicles (Fig. 2f). In addition, there was thickening of the lining of the uterus, apoptotic necrosis, and distension of the endometrium (Fig. 3b and c). The results suggested that regulated pathophysiologic processes were induced during a primary and repeat genital chlamydial infection.

### Pattern and number of differentially expressed miRNAs are different after a primary *C. muridarum* infection and reinfection and is time dependent

**Fig. 4:**
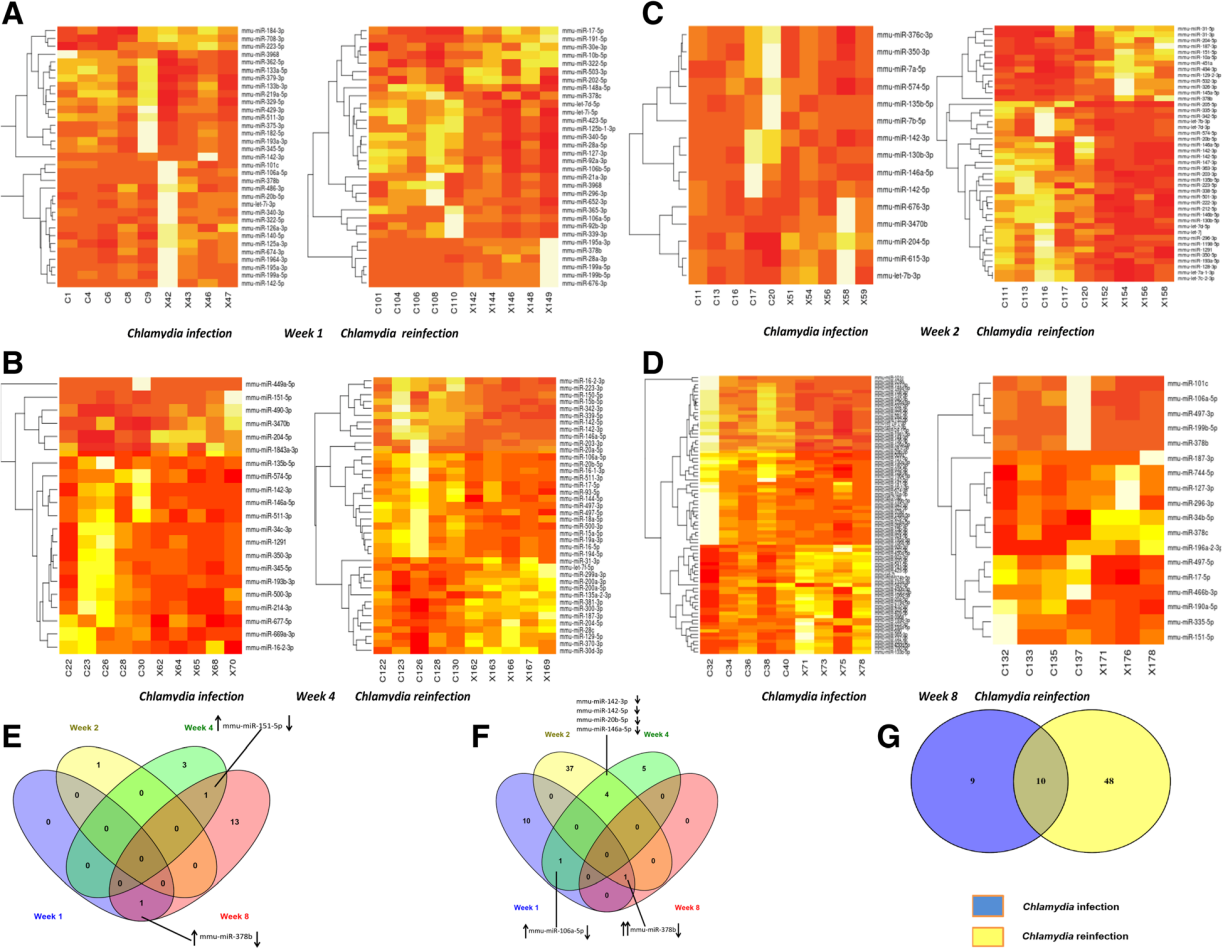
Heat map and Cluster analysis showing the numbers of differentially expressed miRNAs after chlamydia infection and reinfection. **a**. Heat map showed that there is a difference in miRNA expression profile 1 week after chlamydia infection and reinfection. R script was used to create the heatmap for each set of sample comparison. **b**. Heat map showed that there is a difference in miRNA expression profile 2 weeks after chlamydia infection and reinfection. R script was used to create the heatmap for each set of sample comparison. **c**. Heat map showed that there is a difference in miRNA expression profile 4 weeks after chlamydia infection and reinfection. R script was used to create the heatmap for each set of sample comparison. **d**. Heat map showed that there is a difference in miRNA expression profile 8 weeks after chlamydia infection and reinfection. R script was used to create the heatmap for each set of sample comparison. **e**. Venn diagram of differentially expressed miRNAs after chlamydia infection. Figure compares the differentially expressed microRNAs 1, 2, 4 and 8 weeks after infection. The numbers in the Venn diagram represents the number of distinct and common microRNAs in the different weeks of infection. There were no common microRNAs expressed in all weeks of infection. *p*-values here were adjusted by false discovery rate. **f**. Venn diagram of differentially expressed miRNAs after chlamydia reinfection. Figure compares the differentially expressed microRNAs 1, 2, 4 and 8 weeks after infection. The numbers in the Venn diagram represents the number of distinct and common microRNAs in the different weeks of infection. There were no common microRNAs expressed in all weeks of infection. p-values here were adjusted by false discovery rate. **g**. Venn Diagram comparing miRNAs expressed during chlamydia infection and reinfection. Figure compares all the differentially expressed microRNAs in chlamydia infection and reinfection. The numbers in the Venn diagram represents the number of distinct and common microRNAs. There were 10 common microRNAs expressed in both chlamydia infection and reinfection. p-values here were adjusted by false discovery rate

We investigated the role of miRNAs in regulating the pathophysiologic processes induced during a primary and repeat genital chlamydial infection. We analyzed the miRNA sequencing data and observed that there were more miRNA differentially expressed in reinfection compared to single infection, however in both cases more miRNAs were down regulated during *C. muridarum* infection than were upregulated (Fig. 4a-g; Tables 1, 2, 3). The number of miRNAs that were differentially expressed varied based on the time after infection when the samples were collected. After a single infection, the highest number of differentially expressed miRNAs was observed in week 8. However, after reinfection weeks 1 and 2 had the highest number of differentially expressed miRNAs (Fig. 4e and f; S. Figs. 1 and 2; Table 1). To eliminate any chance of having false positives in the miRNA analysis, we performed a false discovery rate (FDR) analysis. This analysis reduced the number of miRNAs that were significantly differentially expressed. In all, 21 miRNAs in total were significantly expressed after a single infection, most of these miRNAs have human homologs The log fold changes ranged from − 4.83 to 2.93. Some miRNAs appeared more than once (mmu-miR-378b and mmu-miR-151-5p). miR-3470b was the most upregulated miRNA, while mmu-miR-378b was the most down regulated miRNA. Interestingly, mmu-miR-378b was upregulated in week 1 after infection and then downregulated in week 8 (Fig. 4e; Table 2). After FDR corrected analysis we obsevered that 66 miRNAs were significantly expressed during chlamydia reinfection, most of these miRNAs have human homologs. Six of the miRNAs; mmu-miR-378b, mmu-miR-106a, mmu-miR-142-3p, mmu-miR-142-5p, mmu-miR-20b-5p, mmu-miR-146a-5p; appeared more than once. Log fold changes ranged from − 5.14 to 3.19 (Fig. 4f; Table 3). mmu-miR-378b was the most upregulated miRNA, while mmu-miR-205-5p was the most down regulated miRNA (Table 3). Similar to what we observed after a single infection, mmu-miR-378b fold change value decreased as the infection progressed from week 1 to week 8. Ten miRNAs were found to be common in chlamydia infection and reinfection. These microRNAs are mmu-miR-378b, mmu-miR-204-5p, mmu-miR-151-5p, mmu-miR-142-3p, mmu-miR-128-3p, mmu-miR-335-3p, mmu-miR-195a-3p, mmu-miR-142-5p, mmu-miR-106a-5p and mmu-miR-92a-3p (Fig. 4g). The results suggested that these changes in miRNAs might be associated with the pathological changes that we had observed in the primary *C. muridarum* infection and reinfection, there is a need to understand what pathways they control.

**Table 1:** Number of differentially expressed miRNAs in chlamydia infection and reinfection

	Week 1	Week 2	Week 4	Week 8	Total
Chlamydia infection *P* < 0.05	1	1	4	15	21
Up regulation	1	1	2	7	11
Down regulation	0	0	2	8	10
Chlamydia reinfection *P* < 0.05	12	42	10	1	65
Up regulation	7	13	0	0	20
Down regulation	5	29	10	1	45

miRNAs expressed weeks 1, 2, 4 and 8 after infection. P-values were adjusted or controlled by false discovery rate. Upregulation indicates positive fold change of the expressed miRNAs while downregulation indicates negative fold change of the expressed miRNAs

**Table 2:** miRNAs differentially expressed after Chlamydia infection

Week	miR	Fold Change(Log)	*P* value*	Direction
Week 1	mmu-miR-378b^a^	2.91	0.000057	UP
Week 2	mmu-miR-3470b^c^	2.93	0.000003	UP
Week 4	mmu-miR-204-5p^a^	1.43	0.000276	UP
	mmu-miR-151-5p^b^	1.83	0.000292	UP
	mmu-miR-142-3p^a^	−1.48	0.000446	DOWN
	mmu-miR-449a-5p^a^	−2.07	0.000493	DOWN
Week 8	mmu-miR-378b^a^	−4.83	0.000000	DOWN
	mmu-miR-128-3p^b^	−2.01	0.000016	DOWN
	mmu-miR-151-5p^b^	−1.78	0.000018	DOWN
	mmu-miR-101c^b^	−3.20	0.000035	DOWN
	mmu-miR-335-3p^a^	2.03	0.000101	UP
	mmu-miR-450b-5p^b^	2.11	0.000108	UP
	mmu-miR-195a-3p^b^	−2.11	0.000222	DOWN
	mmu-miR-92b-3p^a^	1.94	0.000260	UP
	mmu-miR-142-5p^a^	−1.92	0.000319	DOWN
	mmu-miR-106a-5p^a^	−2.14	0.000438	DOWN
	mmu-miR-690^c^	1.67	0.000692	UP
	mmu-miR-148a-3p^a^	−1.36	0.001044	DOWN
	mmu-miR-450a-5p^a^	2.19	0.001104	UP
	mmu-miR-133b-3p^b^	1.63	0.001430	UP
	mmu-miR-92a-3p^a^	1.75	0.001695	UP

miRNAs expressed weeks 1, 2, 4 and 8 after infection; ^a^ miRNAs that have an exact human homolog in miRBase; ^b^ miRNAs that do not have an exact human homolog in miRBase, but might be different by a few nucleotides, ^c^ no known human homolog in miRBase. *P-value significance threshold was set at p < 0.05. p-values here were adjusted or controlled by false discovery rate. Positive fold change (log) value indicates upregulation of the expressed miRNA while a negative fold change (log) value indicates downregulation of the expressed miRNA

**Table 3:** Differentially expressed miRNAs after Chlamydia reinfections

Week	miR	Fold Change(Log)	*P* value*	Direction
Week 1	mmu-miR-378b^a^	3.19	0.0000009	UP
	mmu-miR-3968^c^	−1.75	0.000042	DOWN
	mmu-miR-106a-5p^a^	1.86	0.000091	UP
	mmu-miR-423-5p^a^	−1.52	0.00016	DOWN
	mmu-miR-92a-3p^a^	−1.70	0.00022	DOWN
	mmu-miR-199a-5p^a^	1.78	0.000257	UP
	mmu-miR-127-3p^a^	−1.35	0.000332	DOWN
	mmu-miR-17-5p^a^	1.20	0.000495	UP
	mmu-miR-340-5p^a^	−1.26	0.000698	DOWN
	mmu-miR-195a-3p^b^	1.25	0.001594	UP
	mmu-miR-322-5p ^c^	1.30	0.00161	UP
	mmu-miR-199b-5p^a^	1.47	0.001765	UP
Week 2	mmu-miR-205-5p^a^	−5.14	0.0000000	DOWN
	mmu-miR-342-5p^a^	−3.18	0.0000000	DOWN
	mmu-miR-147-3p^b^	−3.29	0.0000000	DOWN
	mmu-miR-135b-5p^a^	−2.06	0.0000000	DOWN
	mmu-miR-363-3p^a^	−2.55	0.000001	DOWN
	mmu-miR-142-3p^a^	−2.46	0.000001	DOWN
	mmu-miR-145a-5p^b^	2.03	0.000005	UP
	mmu-miR-142-5p^a^	−1.81	0.000012	DOWN
	mmu-miR-129-2-3p^a^	1.74	0.000013	UP
	mmu-miR-187-3p^a^	1.50	0.000039	UP
	mmu-miR-451a^a^	1.43	0.000105	UP
	mmu-miR-31–3p^a^	2.07	0.00013	UP
	mmu-miR-326-3p^a^	1.48	0.000185	UP
	mmu-miR-204-5p^a^	1.41	0.000188	UP
	mmu-miR-532-3p^a^	1.54	0.000195	UP
	mmu-miR-146b-5p^b^	−1.34	0.000219	DOWN
	mmu-miR-146a-5p^a^	−1.74	0.000231	DOWN
	mmu-miR-20b-5p^a^	−1.73	0.000402	DOWN
	mmu-miR-151-5p^b^	1.24	0.000438	UP
	mmu-miR-335-3p^a^	−1.96	0.000541	DOWN
	mmu-miR-212-5p^a^	−1.18	0.000733	DOWN
	mmu-miR-31-5p^b^	1.45	0.000829	UP
	mmu-miR-130b-5p^a^	−1.25	0.000892	DOWN
	mmu-let-7d-3p^a^	−1.32	0.001096	DOWN
	mmu-let-7b-3p^a^	−1.46	0.001203	DOWN
	mmu-miR-378b^a^	1.77	0.001282	UP
	mmu-miR-350-5p ^c^	−1.33	0.00141	DOWN
	mmu-miR-1198-5p ^c^	−1.28	0.001676	DOWN
	mmu-let-7j^b^	−1.24	0.00175	DOWN
	mmu-miR-222-3p^a^	−1.01	0.001753	DOWN
	mmu-miR-574-5p^a^	−1.64	0.001819	DOWN
	mmu-miR-128-3p^b^	−1.05	0.00218	DOWN
	mmu-let-7a-1-3p^a^	−1.06	0.002268	DOWN
	mmu-let-7c-2-3p^a^	−1.06	0.002284	DOWN
	mmu-miR-203-3p^b^	−0.98	0.003381	DOWN
	mmu-miR-1291^a^	−1.11	0.00341	DOWN
	mmu-miR-10a-5p^a^	0.96	0.003667	UP
	mmu-miR-338-5p^a^	−1.14	0.004249	DOWN
	mmu-miR-223-5p^a^	−1.22	0.004819	DOWN
	mmu-miR-193a-5p^a^	−1.10	0.005228	DOWN
	mmu-miR-494-3p^a^	1.04	0.005263	UP
	mmu-miR-296-3p^a^	−1.16	0.005263	DOWN
Week 4	mmu-miR-142-3p^a^	−2.17	0.0000000	DOWN
	mmu-miR-106a-5p^a^	−1.77	0.000001	DOWN
	mmu-miR-142-5p^a^	−1.42	0.000048	DOWN
	mmu-miR-339-5p^a^	−1.20	0.000052	DOWN
	mmu-miR-20b-5p^a^	−1.42	0.000055	DOWN
	mmu-miR-146a-5p^a^	−1.49	0.000209	DOWN
	mmu-miR-511–3p^a^	−1.38	0.000261	DOWN
	mmu-miR-16-2-3p^a^	−1.34	0.000433	DOWN
	mmu-miR-15a-5p^a^	−1.07	0.000485	DOWN
	mmu-miR-497-3p^a^	−1.20	0.001221	DOWN
Week 8	mmu-miR-378b^a^	−3.84	0.000081	DOWN

miRNAs expressed weeks 1, 2, 4 and 8 after infection; ^a^ miRNAs that have an exact human homolog in miRBase; ^b^ miRNAs that do not have an exact human homolog in miRBase, but might be different by a few nucleotides, ^c^ no known human homolog in miRBase. *P-value significance threshold was set at p < 0.05. p-values here were adjusted or controlled by false discovery rate. Positive fold change (log) value indicates upregulation of the expressed miRNA while a negative fold change (log) value indicates downregulation of the expressed miRNAs

### Differentially expressed miRNAs in *C. muridarum* infection regulate myriad pathways involved in disease and biological functions

Core analysis was performed on all miRNAs expressed after single *C. muridarum* infection and reinfection in the context of molecular network and biological processes in mice using the Diana-mirPath and Ingenuity Pathway Analysis (IPA) algorithms. Using Diana-mirPath [30], our analysis showed that for the single infection, 57 pathways were predicted to be regulated by the differentially expressed miRNAs (Additional file 1: Figure S3). The pathways included extracellular matrix-receptor interaction, leukocyte trans-endothelial migration, endometrial cancer, bacterial invasion of epithelial cells, PI3-AKT signaling, mTor signaling, protein processing in the endoplasmic reticulum, and focal adhesion. 61 pathways were predicted to be regulated by the differentially expressed miRNAs after chlamydia reinfection (Additional file 1: Figure S4). The pathways included T and B cell signaling, TGF-beta signaling, glioma, different forms of cancer, adherens junction, endocytosis, RNA degradation, bacterial invasion of epithelial cells, endometrial cancer, FCψ receptor (FCψR)-mediated phagocytosis, Wnt signaling and extracellular matrix (ECM) receptor interactions. More pathways were predicted to be regulated after reinfection due to the higher number of differentially expressed miRNAs, we further analysed our data using IPA, to determine if there were any subtle differences in these pathways in mice.

**Fig. 5:**
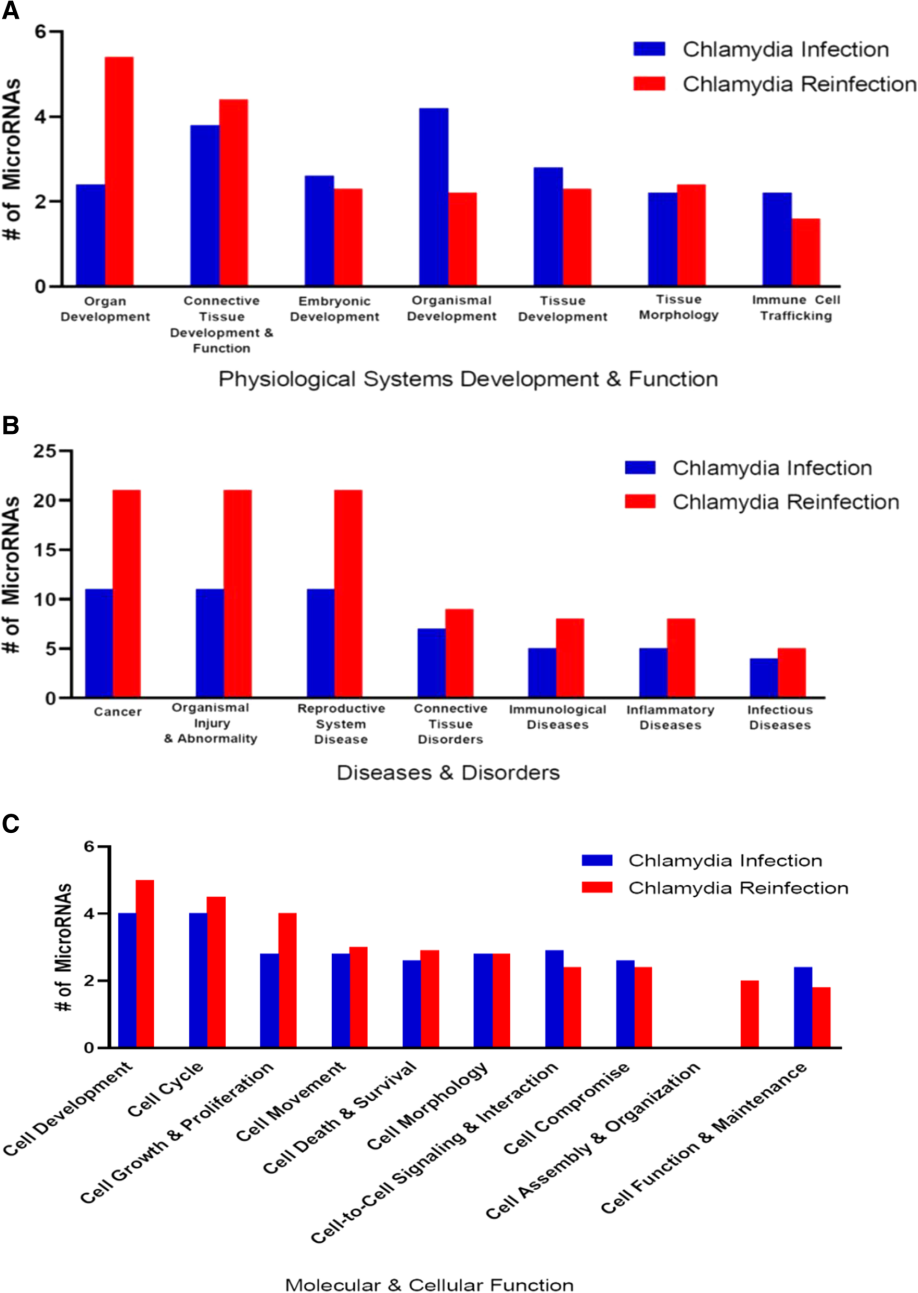
Pathways predicted to be regulated by miRNAs differentially expressed in mice after chlamydia infection and reinfection. **a**. Pathways predicted to regulate physiological system development after chlamydia infection and reinfection. This analysis was determined using Ingenuity pathway analysis software (IPA). miRNAs used in the analysis were from the list derived after FDR correction. **b**. Pathways predicted to regulate diseases and disorders after chlamydia infection and reinfection. This analysis was determined using Ingenuity pathway analysis software (IPA). miRNAs used in the analysis were from the list derived after FDR correction. **c**. Pathways predicted to regulate molecular and cellular functions after chlamydia infection and reinfection. This analysis was determined using Ingenuity pathway analysis (IPA). miRNAs used in the analysis were from the list derived after FDR correction

The IPA core expression analysis provides a rapid assessment of the signaling and metabolic pathways, upstream regulators, molecular interaction networks, and diseases and biological functions that are most likely to be perturbed based on the changes of expression of miRNAs after a single chlamydia infection and reinfection. We focused on four categories when assessing the effects of *C. muridarum* on the differential expression on miRNAs; physiological system development, diseases and disorders, molecular and cellular functions, and toxicity functions. In pathways associated with physiological system development, the results show that there were differences in pathways regulating organ development, organismal development and connective tissue development & function after chlamydia infection and reinfection (Fig. 5a). In diseases and disorders associated pathways, there were differences in pathways regulating tumorigenesis or cancer, organismal injury & abnormalities disease and reproductive system disease after chlamydia infection and reinfection (Fig. 5b). In pathways associated with molecular and cellular functions the results show that there were differences after a single chlamydia infection and reinfection especially in cellular development, cell cycle, and cellular growth and proliferation. (Fig. 5c).

During a single *C. muridarum* infection, the most significant pathways within the physiological systems development and functions category included organismal development and connective tissue development and function (Fig. 5a; Additional file 1: Table S1). The most significant pathways within the diseases and disorders category included cancer, organismal injury and abnormalities disease and reproductive system disease, these pathways had the same number [12] of miRNAs (Fig. 5b; Additional file 1: Table S1). The most significant pathways within the molecular and cellular functions included cellular development, cell cycle and cell-to-cell signaling and interaction (Fig. 5 c; Additional file 1: Table S1).

During *C. muridarum* reinfection, the most significant pathways within the physiological system development and functions category included organ and connective tissue development (Fig. 5a; Additional file 1: Table S1). The most significant pathways within the diseases and disorders category included cancer, organismal injury and abnormalities disease (Fig. 5b; Additional file 1: Table S1). The most significant pathways within the molecular and cellular functions included, cellular development and cellular growth and proliferation (Fig. 5c; Additional file 1: Table S1). We did a further analysis of the regulated pathways and showed precisely the key steps where these miRNAs associated with chlamydia reinfection had been described to play a role (Additional file 1: Table S2).

We evaluated the top networks of miRNAs expressed during *C. muridarum* infection and generated two associated networks using IPA knowledge base. Network 1 consists of miRNAs and molecules associated with cancer, organismal Injury and abnormalities and reproductive system disease. We observed 10 focus molecules. The transcription factor CCAAT/Enhancer Binding Protein Alpha (CEBPA), was the central focus molecule. Network 2 consists of miRNAs and molecules associated organismal injury and abnormalities, reproductive system disease and cancer. There were 6 focus molecules. The transcription factor CAMP Responsive Element Binding Protein 1 (CREB1), was the central focus molecule (Additional file 1: Figure S5A & B).

We further evaluated the miRNAs networks expressed during *C. muridarum* reinfection and generated five associated network using IPA knowledge base (Additional file 1: Figure S5C-G; Additional file 1: Table S2). Network 1 consists of miRNAs and molecules associated with organismal injury and abnormalities, reproductive system disease and cancer. It has 11 focus molecules. The transcription factor Ras Homolog Family Member A (RHOA), was the central focus molecule. Network 2 consists of miRNAs and molecules associated organismal injury and abnormalities, reproductive system disease and cancer. It has 6 focus molecules. Mmu-miR-423-5p, was the central focus molecule (Additional file 1: Figure S5C-G, Additional file 1: Table S2). The results suggested that the miRNAs expression pattern after *C. muridarum* infection plays a role in the pathophysiologic processes associated with the infection.

## Discussion

Understanding the role of host factors in the chlamydial pathogenesis is important in diagnosing infection associated complications and developing new treatments for them. In this study, we determined changes in miRNA expression after chlamydia infection and predicted the role miRNAs might play in disease outcome. Several studies have elucidated the role of miRNAs during chlamydia infection [10, 13, 17, 18, 22–26, 31]. These studies show that miRNAs are important factors in host cell changes and response during chlamydial infection. However, none of the studies on mice genital chlamydial infection have defined the full complement of miRNAs found within the genital tract. The current study was designed to sequence all the known miRNAs found within mice genital tract during chlamydial infection and reinfection. The results reported here are based on all known miRNAs found in the mouse genome. We determined the miRNA expression profiles at different time points after infection; from week 1 to 8. This was done to follow miRNAs progression with time and determine if the changes in miRNAs expression will coincide with pathological changes identified in the mice genital tract.

Histopathological results showed that there was a significant difference in uterine tissue alterations between chlamydia infected and reinfected infected mice. Mice infected once with chlamydia had eosinophilic changes in the endometrium with lymphocytic inflammation associated with apoptotic necrosis, and these features increased over time. The uterus from chlamydia reinfected mice had increased tissue alterations such as formation of new fibrous tissues associated with inflammation, thickening of the lining of the uterus, apoptotic necrosis, and distension of the endometrium. However, there was no apparent association of the occurrence of these characteristics with time, although the the thickening of the endometrial lining was more prominent in the reinfection at week 8. This indicates that after the first infection, pathological changes increased in severity during the reinfection. This is similar to the reported pathologies in women, with increase in complications associated with chlamydia infection occurring in recurrent infections [32].

Here we provide a further insight into the role played by miRNAs during genital chlamydial infection by providing miRNAs associated with disease progression. In addition, we depict the possible pathways associated with the differential expression of these miRNAs during chlamydial infection. In this study, we utilized next generation sequencing (NGS); the best method for determining the miRNAs expression. NGS has several advantages over microarray or quantitative (q) PCR-based miRNA expression profiling techniques. This includes, high sensitivity to determining miRNA levels over a wide dynamic range, ability to identify novel miRNAs and to detect miRNA expression levels in species for which complete genomes are not yet available. In addition, NGS can detect miRNAs that differ by just one nucleotide. Multiplexing of samples by tagging libraries with barcodes during library preparation is possible in NGS. However, NGS is expensive even though the price is decreasing [33, 34]. The sequences identified from the NGS were subsequently analyzed using the comprehensive analysis pipeline for miRNA-sequence data (CAP-miRSeq), which is a powerful and flexible tool used to process and analyze miRNA-seq data [35]. This study offers evidence that miRNAs differentially expressed during chlamydia infection and reinfection differ in number, types and regulatory pathways in a time dependent manner.

Initial cluster analysis show a difference in miRNA expression during chlamydia infection and reinfection at weeks one, two, four and eight after chlamydial infection. Other studies have looked at earlier time points (1 week to 12 days) of a single infection [17, 18, 25] or only during a second infection [10]. Here we report that there is a difference in the miRNA signature based on the number of chlamydial infections. During single chlamydia infection, 11 miRNAs were up-regulated while 10 miRNAs were down-regulated. During the chlamydia reinfection, 20 miRNAs were up-regulated while 45 miRNAs were down-regulated. We have shown in previous studies that more miRNAs are downregulated during genital chlamydia infection than are upregulated, and we related these changes in differential expression to increased expression of genes associated with fibrosis, epithelial to mesenchymal transition, cell proliferation, growth and differentiation [10, 36]. Gupta et al., also showed that several miRNAs are down regulated during chlamydia infection [18]. However, in ocular chlamydia infection there appears to be more upregulation of miRNAs than down regulation [23]. Our findings confirm that there is a alteration in the miRNAs profile during chlamydia infection and reinfection. In addition, we provide information on the miRNA profile after chlamydia infection and reinfection over an extended period (8 weeks). Our data shows that more miRNAs were differentially expressed in the 8 week of the first infection and the second week of reinfection. This correlated with the progression of pathological changes which we had noticed during the first infection. In the repeat infection, after the second week, we did not notice appreciable changes in pathology. This implies an association between pathology and the number of differentially expressed miRNAs at those timepoints. Selection of miRNAs based on FDR has given us an insight into the miRNAs associated with pathogenesis caused by *Chlamydia*. Thus we can imply that the difference in miRNA expression profile and number during chlamydia infection and reinfection could be as a result of regulation through the quick induction of adaptive and protective immunity against *Chlamydia* [27, 37].

Pathway analysis shows that miRNAs expressed in chlamydia infection and reinfection are involved in regulating several pathways from extracellular matrix-receptor interaction, leukocyte trans-endothelial migration, bacterial invasion of epithelial cells, phosphoinositide 3-kinase - protein kinase B (PI3-AKT) signaling, mammalian target of rapamycin (mTor) signaling, protein processing in the endoplasmic reticulum, focal adhesion, T and B cell signaling, Transforming growth factor beta (TGF-beta) signaling, glioma, different forms of cancer, adherens junction, endocytosis, RNA degradation, FCψR-mediated phagocytosis and Wnt signaling. Chlamydia reinfection had more pathways because more miRNAs were differentially expressed after repeat infection. Using IPA we were able to discern differences in networks between chlamydia infection and reinfection involving the cancer, organismal Injury and abnormalities and reproductive system disease, where the networks had different focal molecules. Indicating that the pathogenesis in chlamydia infection and reinfection follow different paths.

Use of miRNAs to understand the pathogenesis of *Chlamydia* is important, our results have some similarities with work previously done, we observed mmu-miR-142 [23, 25]; mmu-miR-146 [18]; mmu-miR-147 [22]; mmu-miR-199b, mmu-miR-142-3p, mmu-miR-204, mmu-miR-449a-5p, mmu-miR-101, mmu-miR-340 [10]. Our previous study [10] had the closest resemblance to our present data with 5 miRNAs being similar. We had reported miRNAs expressed after a reinfection and our present data shows that more miRNAs are differentially expressed during chlamydia reinfection. However, it should be noted that we had used *Lymphogranuloma venereum* instead of *C. muridarum*. During a single chlamydia infection, we observed two miRNAs (mmu-miR-378b and mmu-miR-151-5p) changed direction in their differential expression. miRNA-378b has been shown to be important in regulating epithelial to mesenchymal transition (EMT), keratinocyte differentiation and proliferation, fibrosis and aromatase expression in the genital tract [38–45]. miR-151-5p regulates tumor cell migration and spread; it is blocked by Rho GDP dissociation inhibitor alpha (Rho GDI alpha), and associated with the focal adhesion kinase gene [46, 47]. It positively regulates cell apoptosis by targeting the expression of tumor necrosis factor superfamily member 10 (TNFSF10) and fibronectin type III domain containing 1 (FNDC1) in mice with premature ovarian failure [48]. The increased pathology including increased migration of eosinophils/neutrophils and lessions that increased with time after single chlamydia infection might be associated with the expression of mmu-miR-378b and mmu-miR-151-5p. During chlamydia reinfection, the formation of new fibrous tissues associated with inflammation, thickening of the lining of the uterus, apoptotic necrosis, and distension of the endometrium noticed 4 weeks after infection might be associated with the down regulation of mmu-miR-378b, mmu-miR-146a-5p, mmu-miR-20b-5p, mmu-miR-142-5p, mmu -miR-142-3p and mmu-miR-106a-5p. These miRNAs have been associated with EMT and fibrosis [49–54], which we have hypothesized and shown as being important in the damage to the genital tract during chlamydia pathogenesis [10, 13, 36]. These miRNAs are also associated with cancer [49–52, 54], recently a relationship between chlamydia and ovarian cancer was described [55]. In addition we observed that miR-204-5p, miR-151-5p, miR-378b, miR-142-3p, miR-128-3p, miR-335-3p, miR-142-5p, miR-106a-5p, miR-195a-3p and miR-92a-3p were common in both infections. miR-204 targets Janus kinase 2 (JAK2), and regulates epithelial to mesenchymal transition by targetting Specificity Protein 1 (SP1) in tubular epithelial cells; regulates protein kinase RNA-like endoplasmic reticulum kinase (PERK) and unfolded protein response [56–58], which we have shown to involved in chlamydial pathogenesis [59]; miR-106a-5p suppress the proliferation, migration and invasion of osteosarcoma cells, and is a good predictor of prognosis in hepatocellular cancer [60, 61]; miR-195a-3p targets matrix metalloproteinase 2 (MMP2) in murine mesenchymal stem cells thus inhibiting angiogenesis [62]. MMP2 expression in wild type mice oviduct increases by day 7 after chalmydia infection [63]. miR-92a-3p is significantly expressed in cervical cancer tissue cell lines thus promoting cervical cancer cell proliferation, cell cycle transition from gap 1 (G1) to synthesis (S) phase and invasion [64]. Intrestingly, it has been reported in dual RNA-Seq analyses of in vitro chlamydia infected epithelial cells, that infected epithelial cells expressed aberrant immune dampening and fibrotic responses, which included increased expression of TGF-beta, MMP2 and other ECM proteins [65]. Our results show that the miRNAs identified in this study control the expression of genes linked to chlamydial pathogenesis. Therefore, we speculate that we can use these miRNAs as indicators of chlamydial pathogeneses and maybe even a biomarker for disease leading to PID and infertility. It should be pointed out that the literature is strewn with cancer associated miRNAs and it is possible that associations with cancer noticed in this study maybe cursory.

## Conclusion

The findings from our study show that chlamydial infection induces differentially expressed miRNAs that play a significant role in organismal injuries and abnormalities, cellular development, reproductive disorders, cell cycle, inflammatory response, fibrosis, cancer and connective tissue disorders. The targets and pathway analyses indicate that specific miRNAs are involved in the primary infection and reinfection, although there were overlaps. In addition, our results are corroborated by previous human research findings that have established an association between recurrent or persistent endocervical chlamydial infection in women and an increased risk of pelvic inflammatory disease and scarring sequelae in the upper genital tract, which have also been demonstrated in several animal models [2, 32]. This study highlights the need to further study the association of genital *Chlamydia* infection with the promotion of cancer in women, because several miRNAs in this study having been associated with cancer in other studies [46, 50, 60, 61, 64, 66]. The highly interconnected networks of miRNAs generated from our data shows that there are differences in the focus molecules involved in significant biological functions during chlamydia infection and reinfection, implying that chlamydial pathogenesis may be distinct for each type of infection and that this could be important when determining treatments regime and disease outcome. Despite the strengths of our study, involving: a) next generation sequencing, b) a single infection and reinfection, and c) sample collection up to 8 weeks after infection, it has some limitations. We did not carry out a third infection or assess the effects of antibiotics treatment on each infection, all of which may play a role in miRNA expression and pathogenesis in human infections. Although only certain aspects of the pathogenesis of chlamydial infection in the mice model mirror the human infection [2], our findings show the importance of miRNAs regulation and expression in chlamydial infection and pathogenesis, which has implications for humans. In summary, our study findings provides substantial evidence that miRNAs regulate the host responses and biological processes that are associated with *Chlamydia* pathogenesis.

## Methods

### Ethics statement

The Institutional Animal Care and Use Committee of Morehouse School of Medicine (MSM-IACUC) approved the animal care and use protocol (# 16–24), which we followed in this study. MSM-IACUC adheres to the Natinal Institute of Health (NIH) guidelines for the care and use of laboratory animals, with Public Health Service (PHS) policy, and the Animal Welfare Act.

### Chlamydia stocks

Stocks of *Chlamydia muridarum Nigg* (the agent of mouse pneumonitis) used for infections were prepared by propagating elementary bodies (EBs) in McCoy or HeLa cells (Division of Scientific Research, Centers for Disease Control and Prevention, Atlanta), according to standard procedures. *Chlamydia* stock titers were expressed as inclusion-forming units (IFU) per milliliter [67].

### Animals

Female C57BL/6 J wild type mice were purchased from Jackson Laboratory (Bar Harbor, MA), fed food and provided water ad libitum, and maintained in laminar flow racks under pathogen-free conditions with a 12 h light and 12 h dark cycle. The protocols involving mice were approved by MSM-IACUC. For all invasive procedures mice were anesthesized using isoflurane. Mice were placed in a chamber with 2–4% gas concentration of isoflurane in a fume hood for 30 min. Mice were euthanized using carbon dioxide asphyxiation followed by cervical dislocation. This method of euthanasia is recommended by the Panel on Euthanasia of the American Veterinary Medical Association.

### Infectivity

6–8 weeks old Female C57BL/6 [40] were infected and reinfected (interval of 4-weeks) intravaginally with 1 × 10^5^ IFU /mouse of live *C. muridarum* approximately 7 days after administration of 2.5 mg/mouse Depo Provera (medroxy-progesterone Acetate; Pfizer Inc., NY). Control mice [40] were sham infected with sterile PBS approximately 7 days after administration of 2.5 mg/mouse Depo Provera. Mice were determined to be infected by periodic cervicovaginal swabbing and isolation of *Chlamydia* in tissue culture.

### Histopathology

Histopathology of the genital tract associated with chlamydial infection was investigated. 40 mice were infected and reinfected intravaginally with *C. muridarum* (1 × 10^5^ IFU per mouse) approximately 7 days after administration of 2.5 mg/mouse Depo Provera (medroxy-progesterone Acetate; Pfizer Inc., NY). The entire genital tract from the vagina to the ovary was collected from infected and control mice mice at one, two, four and 8 weeks after infection (5 mice per time point) and fixed in 10% formaldehyde. The samples were embedded in paraffin, cut longitudinally into 5 μm sections to include the cervix, uterine horns, oviducts and ovaries as well as the luminal structures, and stained with hematoxylin and eosin. Blinded to group designations, a board-certified pathologist (UBM) evaluated the uteri, oviducts and ovaries for the presence of lesions including, but not limited to inflammation, edema, fibrosis, and luminal distension. Lesions were recorded as either absent or present. If absent (i.e., histologically normal), a score of 0 was assigned. If present, the severity of the lesions was recorded as minimal, mild, moderate, or severe, with severity scores of 1 through 4, respectively. Lesion distribution was recorded as focal, multifocal, or diffuse, with distribution scores of 1, 2, or 3, respectively. A total severity-and-distribution group score was calculated by adding individual distribution and severity scores. Because the group sizes were uneven, an average severity-and-distribution group score was calculated by dividing the total severity-and-distribution score by the numbers of animals in the group. Note that control and infected mice were from the same batch of mice and that each time point had its own control group.

### Small RNA library construction and Illumina small RNA deep sequencing

Genital tract samples were harvested after sacrificing infected and control mice mice at one, two, four and 8 weeks after infection (5 mice per time point) and frozen immediately on dry ice and then transferred to a − 80 freezer. For non-biased recovery of miRNA, total RNA was isolated using the Direct-zol RNA miniprep (Zymo Research, Irvine CA). RNA integrity was assessed using the Agilent Technologies 2100 Bioanalyzer. Samples were submitted to the University of Georgia Georgia Genomic and Bioinformatic Core (GGBC) for library preparation with the the TruSeq Small RNA Library Preparation Kit (Illumina, San Diego, CA). Samples were sequenced on an Illumina NextSeq 500 instrument using a SE75 run High Output flow cell (Illumina, San Diego, CA, USA), inserts used were 25–75 bases. This assay was validated using qPCR (Additional file 1: Figure S7A & B). Note that control and infected mice were from the same batch of mice and that each time point had its own control group.

### Bioinformatics analysis

NGS data analyses were performed by the GGBC. Quality of raw sequence reads was assessed using FastQC [68] (sequencing depth ≥ 4 million reads). The data were mapped to the *M. musculus* mm10 reference genome and to the MirDeep mouse miRNA database for identifying novel and known miRNA’s, respectively, using the CAP-miRSeq pipeline (version 1.1) [35]. The pipeline leverages several software packages for adapter removal and trimming, read alignment and manipulation of sequence alignment map/binary alignment map (SAM/BAM) and fastq/fasta files (Additional file 1: Figure S8) and implements edgeR for differential expression analysis of counts per million (cpm) normalized count data. Sequences with a perfect match or one mismatch were retained for further analysis. Differential miRNA expression profiling included fold difference estimates between different treatment and time course comparisons as well as *P*-values and false discovery rate (FDR) determinations for each differentially expressed species. Target identification of genes putatively regulated by miRNAs was performed using TargetScan [69]. Heatmaps were generated for differentially expressed miRNAs in each set of comparisons using R. Visualization of overlapping expression among miRNAs identified at different infection timepoints was done using Venny 2.0, an interactive Venn diagram tool for comparing name lists of up to 4 genomic datasets [70]. Software parameters and tools can be found in the Additional file 1.

Pathway, Core and Comparison analyses for all the FDR corrected differentially expressed miRNAs irrespective of time points in infected and reinfected mice were analyzed using the Ingenuity Pathway Analysis software (Qiagen Inc.) and Daina MirPath [30]. The top networks, molecular and cellular functions, diseases and disorders and physiological system development and functions were determined using Ingenuity System Interactive Pathway Systems (version 18,488,943). Stringency was set at ‘highly predicted’ and ‘experimentally validated’. The software uses its own internal algorithm and other databases, including TarBase, TargetScan and miRecords, as well as findings published in the literature. Complete reference set for this analysis was carried out using the Ingenuity Knowledge Base (Genes+ Endogenous Chemicals) for all molecules associated with diseases, functions, pathway, or list annotations.

This study focused on the top networks, molecular and cellular functions, diseases and disorders and physiological system development and functions for each infection. Confidence was set at ‘moderately predicted’, ‘highly predicted’ and ‘experimentally validated. A threshold value of –log (*p*-value) between 1.33 and 21.45 was set to identify molecules whose expression was significantly differentially regulated. The right-tailed Fisher’s exact test was used to calculate a p-value determining the probability that each biological function and/or disease assigned to that data set is due to chance alone [71]. IPA library of canonical pathways that were most significant to the infected and reinfected mice microRNA data set were carried out. Comparison analysis, which analyzed the changes in miRNA expression across observations recorded, was also performed. Graphical representation of the networks generated using molecular relationships between molecules and miRNAs expressed were generated using the IPA software. Molecules are represented as nodes, and the biological relationship between two nodes is represented as an edge (line). All edges are supported by at least one reference from the literature, from a textbook, or from canonical information stored in the Ingenuity Pathways Knowledge Base. Nodes are displayed using various shapes that represent the functional class of the gene product.

### MiRNA validation

Quantitative real time (qRT)-PCR was performed to validate differentially-expressed miRNAs discovered through the deep sequencing. cDNA synthesis and qRT-PCR was performed using Exiqon qPCR microRNA assays kits (Exiqon, Woburn, MA) per the manufacturer’s protocol. The cycle threshold (Ct) values and the relative expression of miRNAs in infected and control samples were normalized to U6 miRNA expression. The qRT-PCR data was analyzed using the delta-delta- Ct (ddCT) method [10].

### Statistical analysis

The data derived from non-sequencing experiments were analyzed and compared by performing a 1- or 2-tailed t-test, and the relationship between different experimental groupings was assessed by analysis of variance (ANOVA) using GraphPad Prism 5.0 software (GraphPad Software, Inc., La Jolla, CA). Statistical significance was judged at *P* < 0.05.

## Additional file

Additional file 1: **Figure S1.** Venn diagram of differentially expressed miRNAs after chlamydia infection. **Figure S2.** Venn diagram of differentially expressed miRNAs after chlamydia reinfection. **Figure S3.** Pathways predicted to be regulated by miRNAs differentially expressed in mice after chlamydia infection. **Figure S4.** Pathways predicted to be regulated by miRNAs differentially expressed in mice after chlamydia reinfection. **Figure S5.** A: Network One for *Chlamydia* infection shows CEBPA a transcription regulator as the focus molecule with associated miRNAs. B: Network Two for *Chlam ydia* infection shows CREB1 a transcription regulator as the focus molecule with associated miRNAs. C: Network One for *Chlamydia* reinfection shows RHOA, an enzyme, as the focus molecule with associated miRNAs and other molecules D: Network Two for *Chlamydia* reinfection shows mir-423-5p as the focus molecule with associated miRNAs and other molecules. E: Network Three for *Chlamydia* reinfection shows TNFSF10 as the focus molecule with associated miRNAs and other molecules. F: Network Four for *Chlamydia* reinfection shows the kinase SRC as the focus molecule with associated miRNAs and other molecules. G: Network Five for *Chlamydia* reinfection shows the transporter BCL2 as the focus molecule with associated miRNAs and other molecules. **Table S1.** Summary Analysis of Top Diseases and Biological Functions of miRNA expressed in the Chlamydia Infection and Re-Infection. **Table S2.** Networks for the Top Diseases and Biological Function Category of miRNA expressed in the *Chlamydia* Reinfection. **Figure S6.** Example of quality scoring for miRNA sequencing in this study. **Figure S7.** (A) Validation of miRNA 378b expression after single infection using qPCR, (B) Validation of miRNA 142-5p expression after single infection using qPCR. **Figure S8.** Comprehensive Analysis Pipeline for miRNA-seq data (CAP-miRSeq), adapted from Sun et al., 2014 [35]. (PDF 1710 kb)

## Abbreviations

AKT: Protein kinase B
ANOVA: Analysis of variance
BAM: Binary alignment map
CAP-miRSeq: Comprehensive analysis pipeline for miRNA-sequence data
cDNA: Complementary DNA
CEBPA: CCAAT/Enhancer Binding Protein Alpha
Cpm: Counts per million
CREB1: CAMP Responsive Element Binding Protein 1
Ct: Cycle threshold
ddCt: delta-delta- Ct
EBs: Elementary bodies
ECM: Extra cellular matrix
EMT: Epithelial to mesenchymal transition
FCψR: FCy receptor
FDR: False discovery rate
FIG: Figure
FNDC1: fibronectin type III domain containing 1
G1: Gap 1 stage
GEO: Gene Expression Omnibus
GGBC: Georgia Genomic and Bioinformatics Core
HE: Hematoxylin and eosin
HPV: herpes virus
IACUC: Institutional Animal Care and Use Committee
IFU: Inclusion-forming units
IPA: Ingenuity pathway analysis software
JAK2: Janus kinase 2
miRNA(s): MicroRNA(s)
miRNA-seq: miRNA-sequencing
MMP2: Matrix metallopeptidase 2
MSM: Morehouse School of Medicine
mTor: mammalian target of rapamycin
NCBI: National Center for Biotechnology Information
NGS: Next generation sequencing
NICHD: National Institute of Child Health and Human Development
NIGMS: National Institute of General Medical Sciences
NIH: National Institute of Health
NIMHD: National Institute on Minority Health and Health Disparities
PERK: Protein kinase RNA-like endoplasmic reticulum kinase
PHS: Public Health Service
PI3: Phosphoinositide 3-kinase
PID: Pelvic inflammatory disease
qPCR: Quantitative PCR
qRT-PCR: Quantitative real time PCR
Rho GDI alpha: Rho GDP dissociation inhibitor alpha
RHOA: Ras Homolog Family Member A
RNA: Ribonucleic acid
S: Synthesis stage
SAM: Sequence alignment map
SP1: Specificity Protein 1
TFI: Tubal factor infertility
TGF: Transforming growth factor beta
TNFSF10: Tumor necrosis factor superfamily member 10
