# Supplementary material for: A unique insight into the MiRNA profile during genital chlamydial infection
Source: BMC Genomics. 2019 Feb 18;20:143. doi: 10.1186/s12864-019-5495-6 (PMC6379932; doi:10.1186/s12864-019-5495-6)
Supplement: Supplementary file 1 — Figure S1. Venn diagram of differentially expressed miRNAs after chlamydia infection. Figure S2. Venn diagram of differentially expressed miRNAs after chlamydia reinfection. Figure S3. Pathways predicted to be regulated by miRNAs differentially expressed in mice after chlamydia infection. Figure S4. Pathways predicted to be regulated by miRNAs differentially expressed in mice after chlamydia reinfection. Figure S5. A: Network One for Chlamydia infection shows CEBPA a transcription regulator as the focus molecule with associated miRNAs. B: Network Two for Chlam ydia infection shows CREB1 a transcription regulator as the focus molecule with associated miRNAs. C: Network One for Chlamydia reinfection shows RHOA, an enzyme, as the focus molecule with associated miRNAs and other molecules D: Network Two for Chlamydia reinfection shows mir-423-5p as the focus molecule with associated miRNAs and other molecules. E: Network Three for Chlamydia reinfection shows TNFSF10 as the focus molecule with associated miRNAs and other molecules. F: Network Four for Chlamydia reinfection shows the kinase SRC as the focus molecule with associated miRNAs and other molecules. G: Network Five for Chlamydia reinfection shows the transporter BCL2 as the focus molecule with associated miRNAs and other molecules. Table S1. Summary Analysis of Top Diseases and Biological Functions of miRNA expressed in the Chlamydia Infection and Re-Infection. Table S2. Networks for the Top Diseases and Biological Function Category of miRNA expressed in the Chlamydia Reinfection. Figure S6. Example of quality scoring for miRNA sequencing in this study. Figure S7. (A) Validation of miRNA 378b expression after single infection using qPCR, (B) Validation of miRNA 142-5p expression after single infection using qPCR. Figure S8. Comprehensive Analysis Pipeline for miRNA-seq data (CAP-miRSeq), adapted from Sun et al., 2014 [35]. (PDF 1710 kb) [file 12864_2019_5495_MOESM1_ESM.pdf]

A unique insight into the MiRNA profile during genital chlamydial infection

Supplementary Results

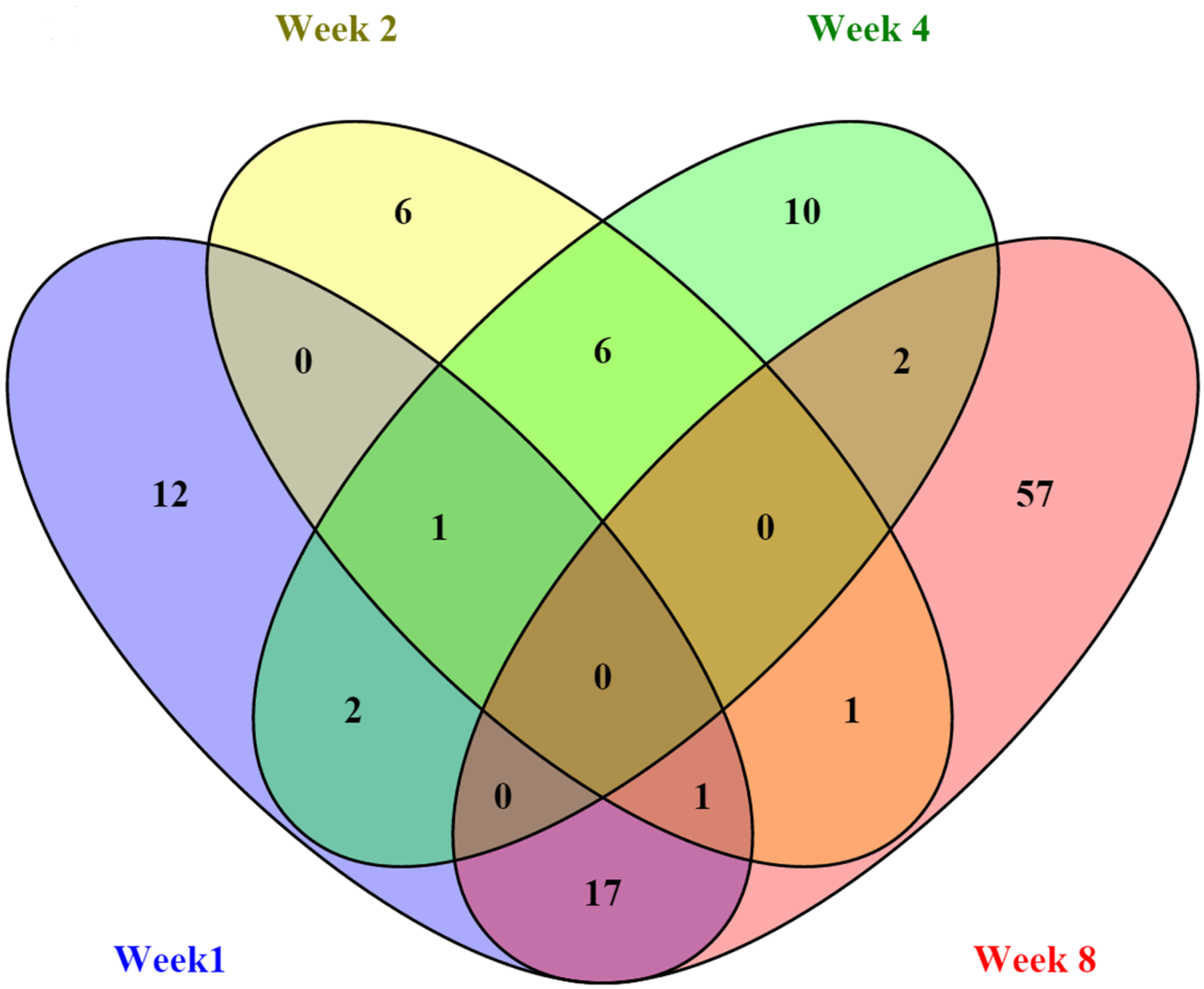

Figure S1: Venn diagram of differentially expressed miRNAs after chlamydia infection. Figure compares the differentially expressed microRNAs 1, 2, 4 and 8 weeks after infection. The numbers in the Venn diagram represents the number of distinct and common microRNAs in the different weeks of infection. There were no common microRNAs expressed in all weeks of infection. p-values here were not adjusted or controlled by false discovery rate.

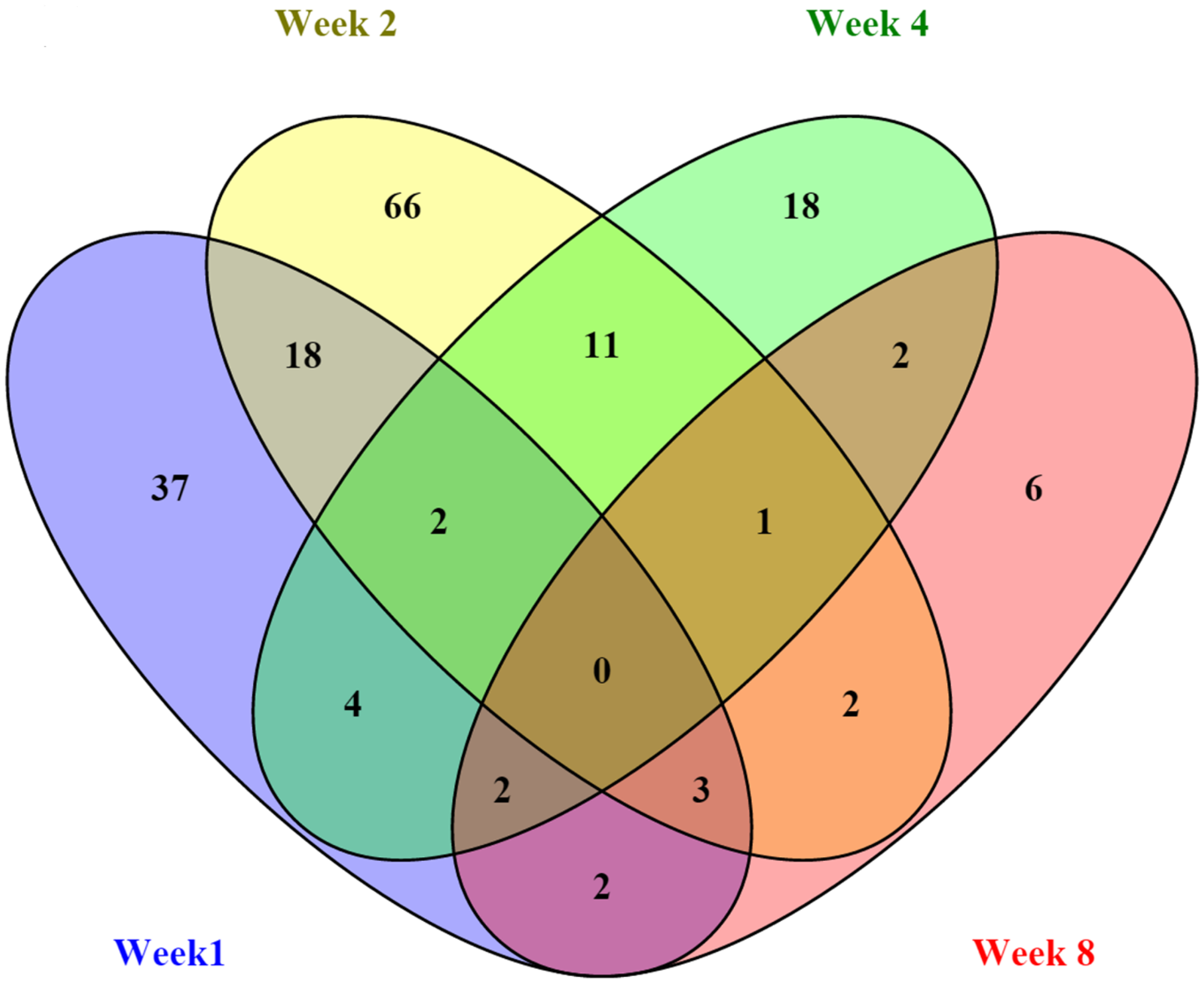

Figure S2: Venn diagram of differentially expressed miRNAs after chlamydia reinfection. Figure compares the differentially expressed microRNAs 1, 2, 4 and 8 weeks after infection. The numbers in the Venn diagram represents the number of distinct and common microRNAs in the different weeks of infection. There were no common microRNAs expressed in all weeks of infection. p-values here were not adjusted or controlled by false discovery rate.

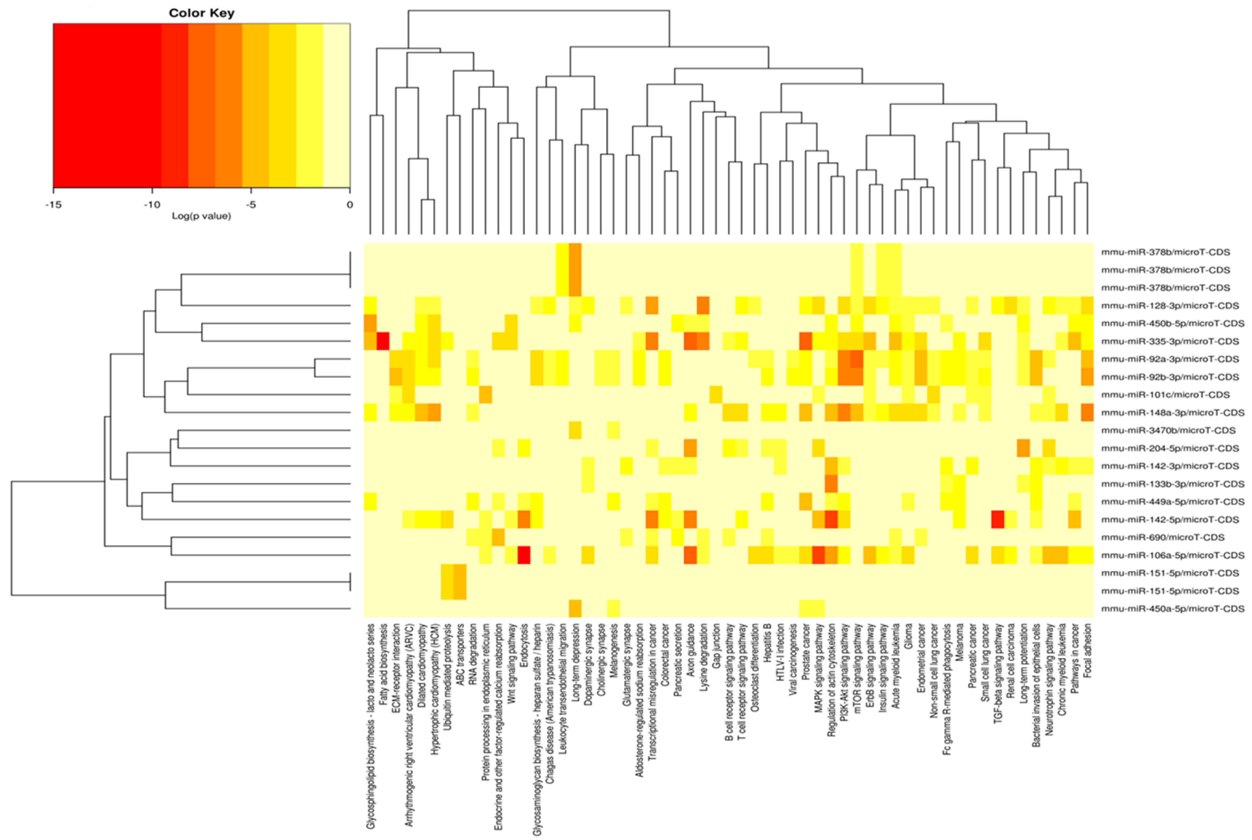

Figure S3: Pathways predicted to be regulated by miRNAs differentially expressed in mice after chlamydia infection. Pathways predicted by differentially expressed miRNAs after chlamydia infection. This analysis was determined using DIANA miRPath v.2.0 program. miRNAs used in the analysis were from the list derived after FDR correction.

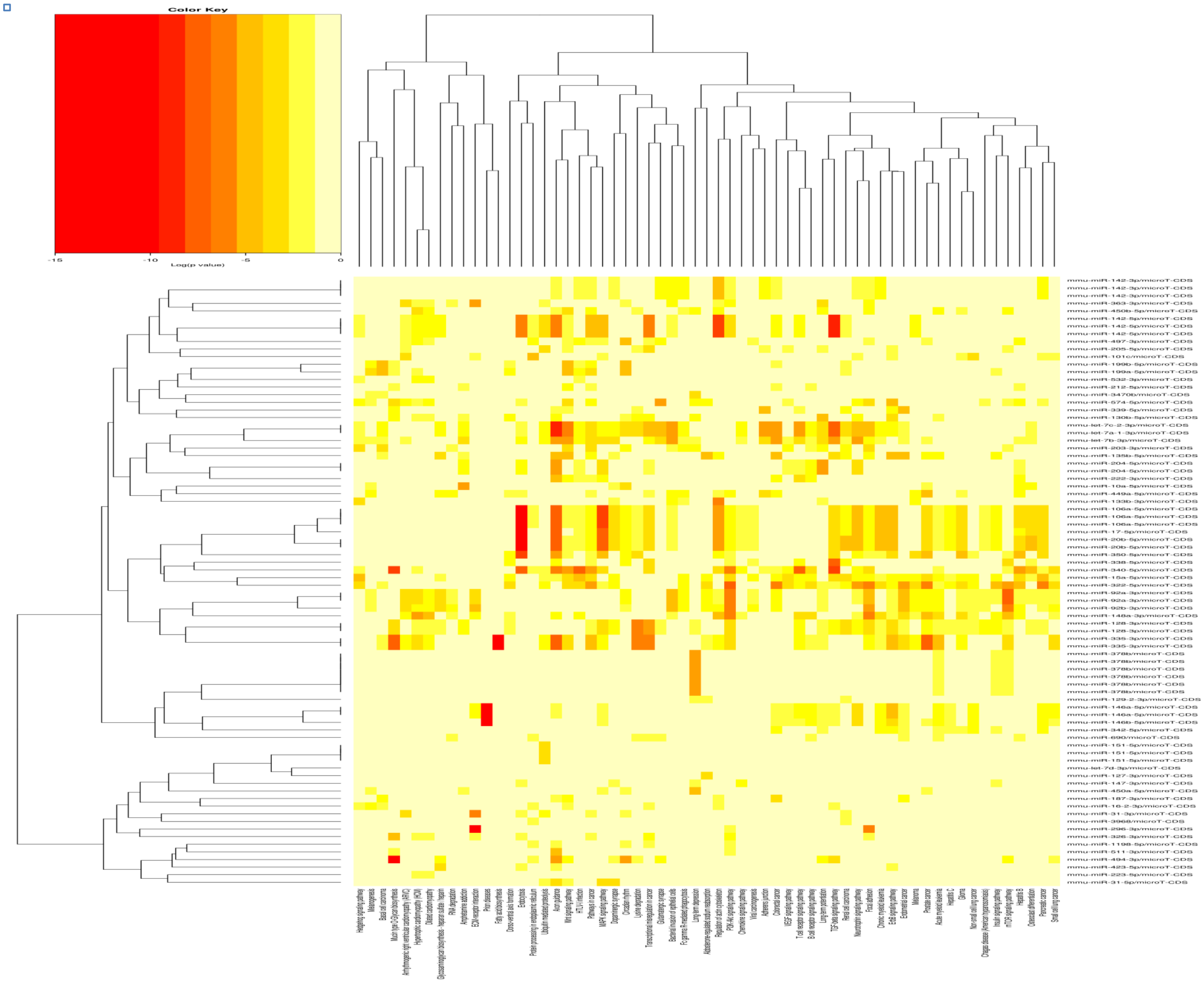

Figure S4: Pathways predicted to be regulated by miRNAs differentially expressed in mice after chlamydia reinfection. Pathways predicted by differentially expressed miRNAs after chlamydia reinfection. This analysis was determined using DIANA miRPath v.2.0 program. miRNAs used in the analysis were from the list derived after FDR correction.

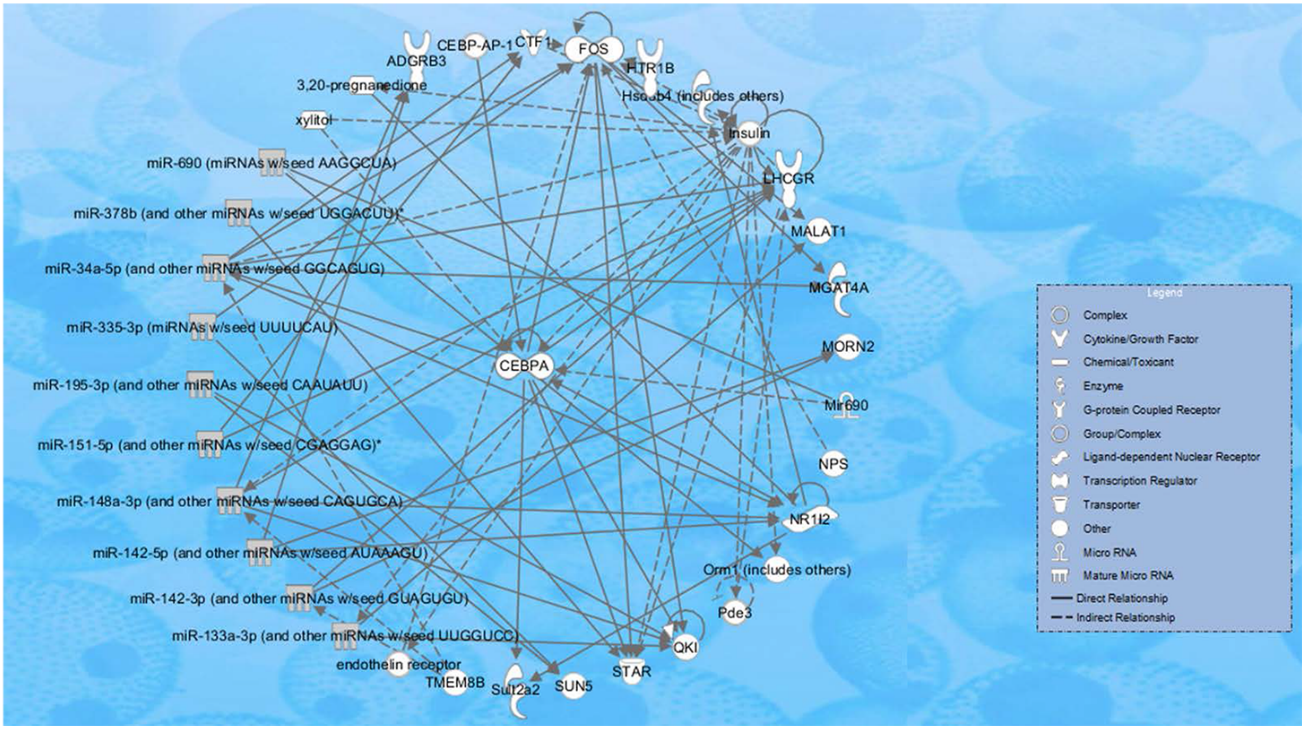

Figure S5A: Network One for *Chlamydia* infection shows CEBPA a transcription regulator as the focus molecule with associated miRNAs.

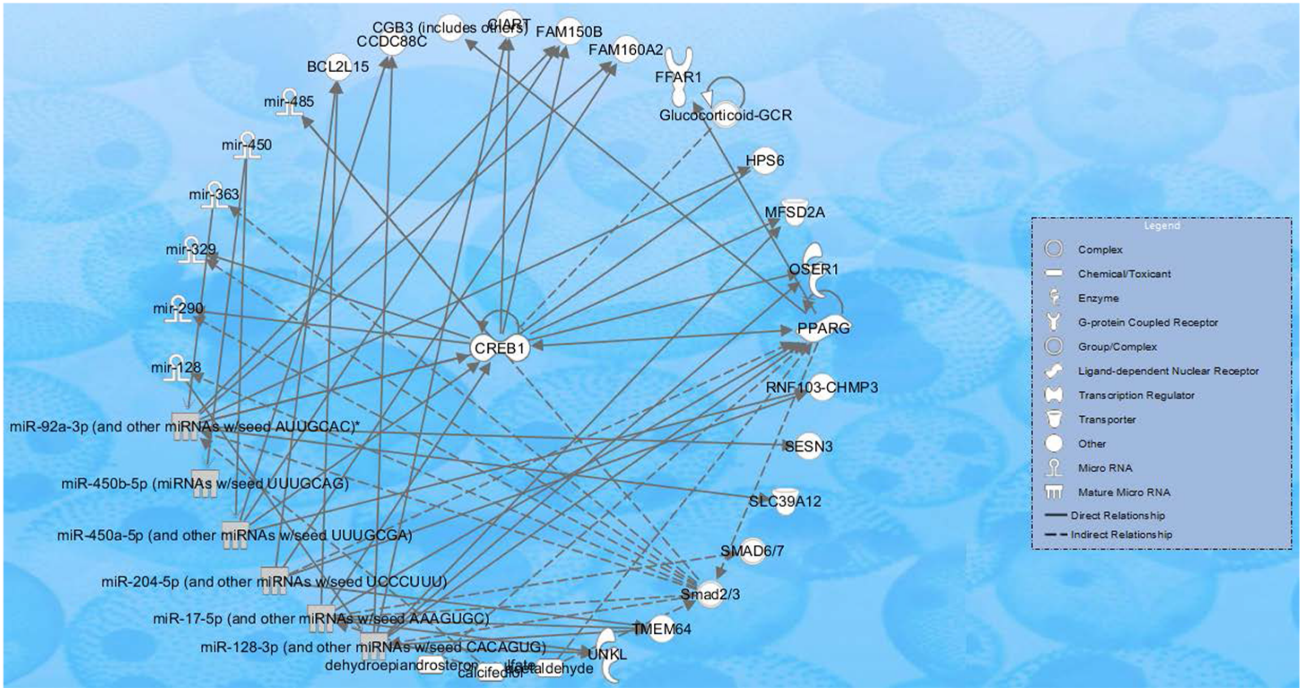

Figure S5B: Network Two for *Chlamydia* infection shows CREB1 a transcription regulator as the focus molecule with associated miRNAs.

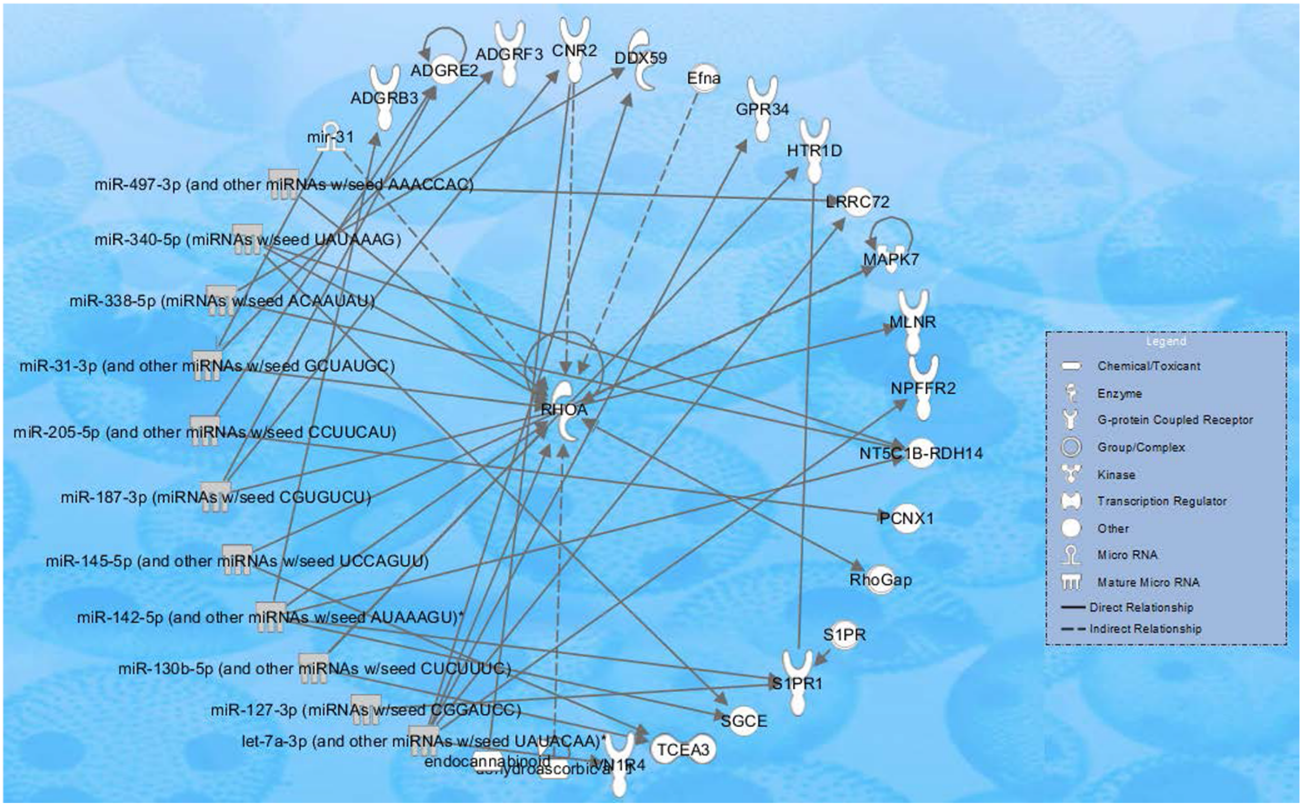

Figure S5C: Network One for *Chlamydia* reinfection shows RHOA an enzyme as the focus molecule with associated miRNAs and other molecules

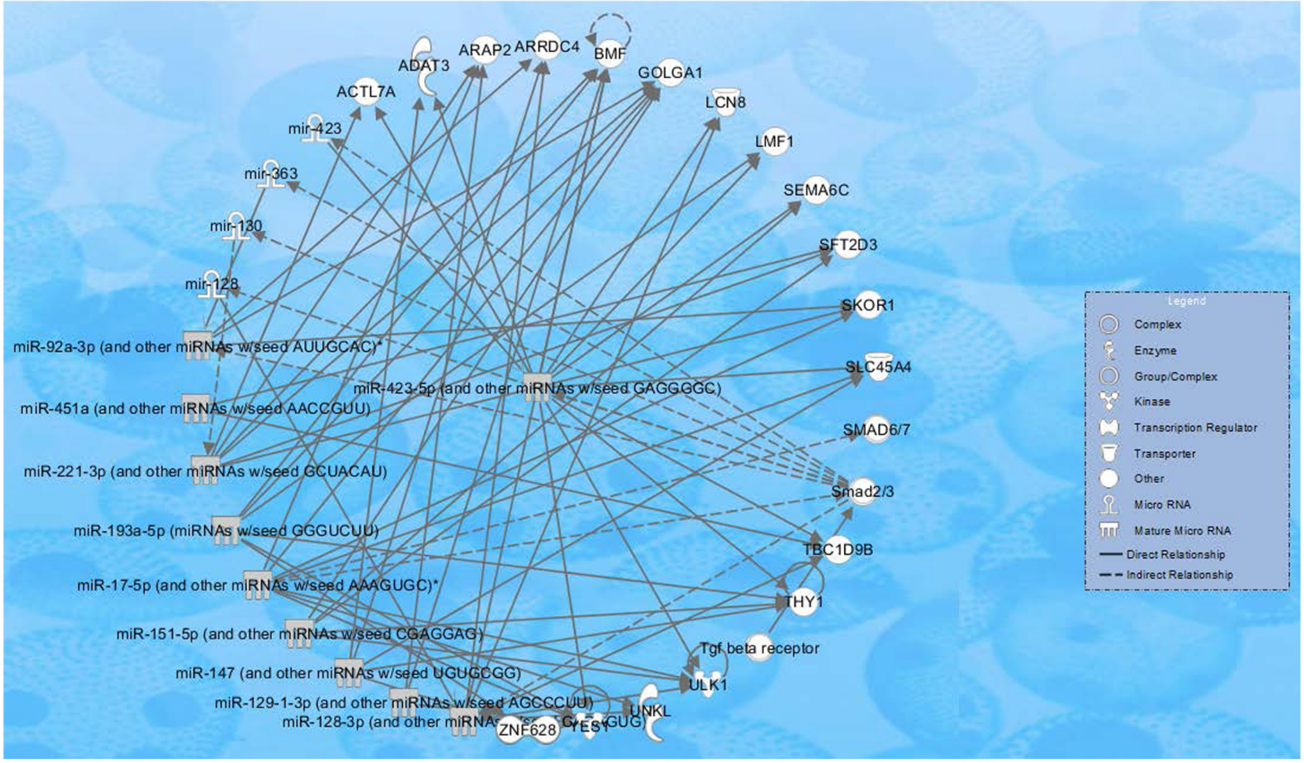

Figure S5D: Network Two for *Chlamydia* reinfection shows mir-423-5p as the focus molecule with associated miRNAs and other molecules

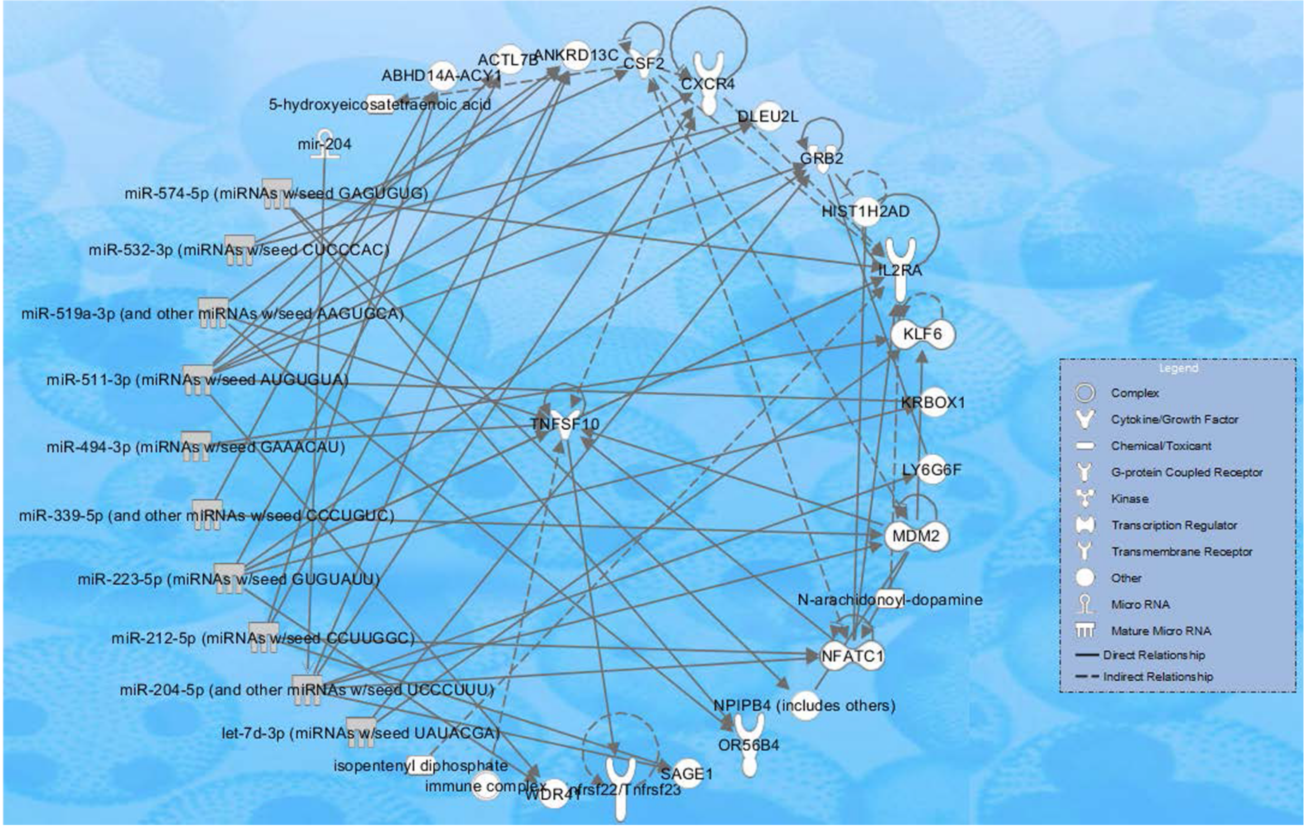

Figure S5E: Network Three for *Chlamydia* reinfection shows TNFSF10 as the focus molecule with associated miRNAs and other molecules

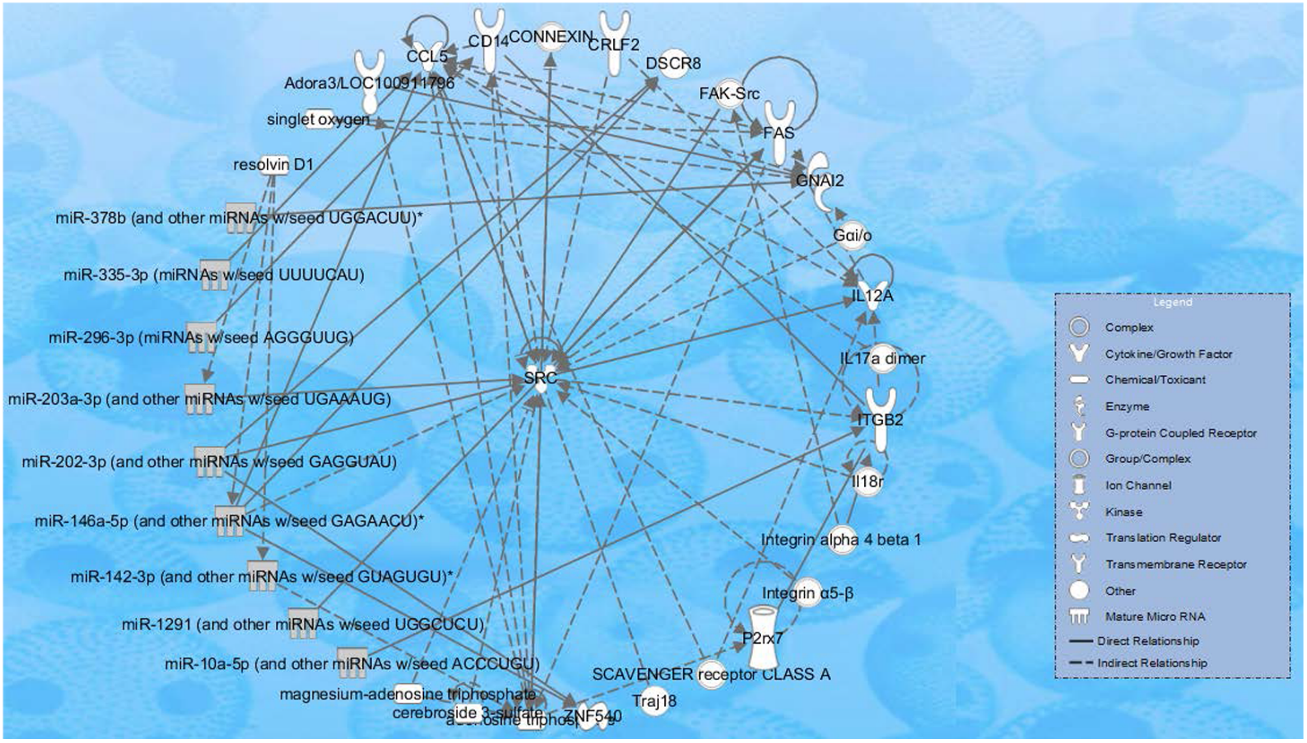

Figure S5F: Network Four for *Chlamydia* reinfection shows the kinase SRC as the focus molecule with associated miRNAs and other molecules

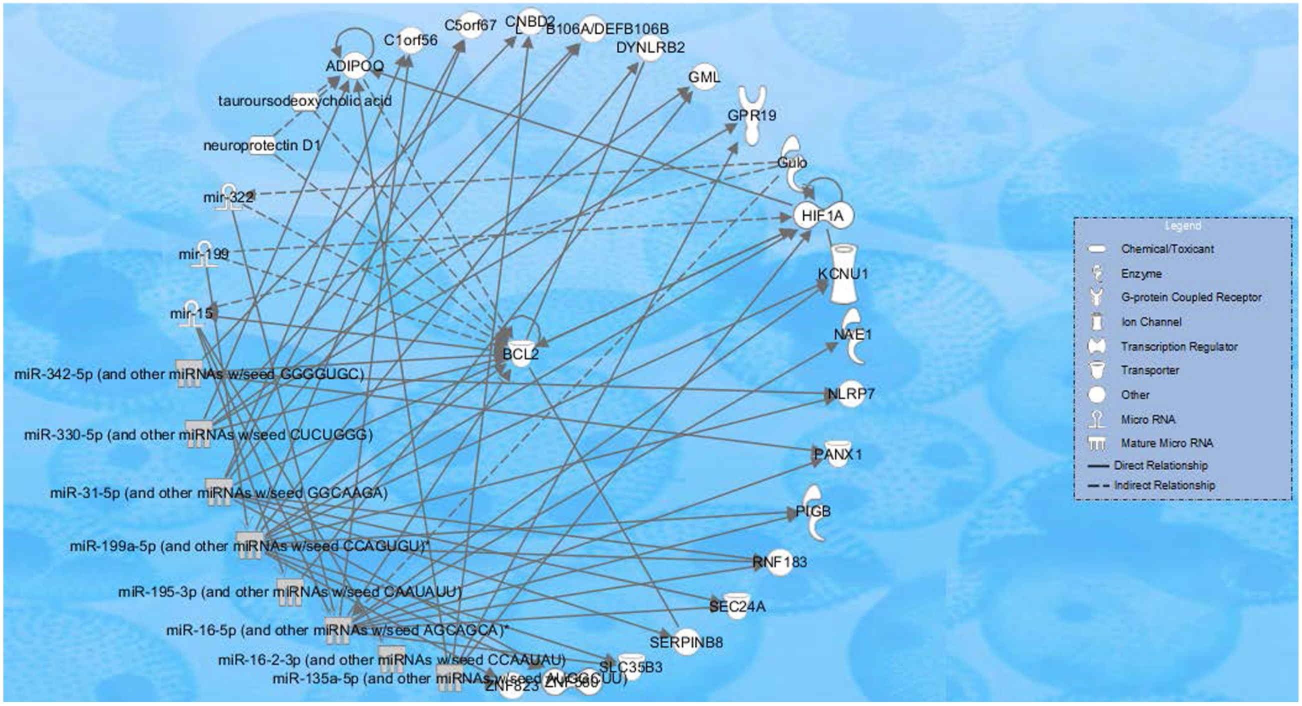

Figure S5G: Network Five for *Chlamydia* reinfection shows the transporter BCL2 as the focus molecule with associated miRNAs and other molecules

Table S1: Summary Analysis of Top Diseases and Biological Functions of miRNA expressed in the Chlamydia Infection and Re-Infection

	Chlamydia Infection		Chlamydia Re-Infection	
**Diseases and Disorders**	**p-value range**	**# of MiRNAs**	**p-value range**	**# of MiRNAs**
**Cancer**	4.96E-02 – 5.34E-12	12	4.92E-02 – 3.51E-22	33
**Organismal Injuries and Abnormalities**	4.96E-02 – 5.34E-12	12	4.92E-02 – 3.51E-22	39
**Reproductive system disease**	3.08E-02 – 5.34E-12	12	3.60E-02 – 3.51E-22	31
**Connective Tissue Disorder**	0	0	7.70E-03 – 2.64E-11	15
**Molecular and Cellular Functions**	**p-value range**	**# of MiRNAs**	**p-value range**	**# of MiRNAs**
**Cellular Development**	4.44E-02 – 4.13E-07	7	4.90E-02 – 3.67E-07	17
**Cell Cycle**	2.82E-02 – 1.81E-04	4	4.16E-02 – 3.50E-05	9
**Cell-To-Cell Signaling and Interaction**	1.40E-02 – 1.22E-03	4	0	0
**Cellular Movement**	2.54E-02 – 1.59E-03	4	4.25E-02 – 5.45E-04	10
**Cell Morphology**	1.83E-03 – 1.83E-03	1	0	0
**Cellular Growth and Proliferation**	0	0	4.90E-02 – 8.95E-05	16
**Cell Death and Survival**	0	0	4.35E-02 – 1.24E-03	9
**Physiological System Development and Function**	**p-value range**	**# of MiRNAs**	**p-value range**	**# of MiRNAs**
**Organismal Development**	4.38E-02 – 4.68E-05	3		
**Connective Tissue Development and Function**	2.22E-04 – 1.81E-04	5	4.90E-02 - 3.50E-05	5
**Tissue Development**	4.38E-02 - 1.22E-03	4	0	0
**Embryonic Development**	4.38E-02 – 1.89E-03	3	0	0
**Organ Development**	0	0	4.67E-02 – 4.08E-06	6

Table S2: Networks for the Top Diseases and Biological Function Category of miRNA expressed in the *Chlamydia* Reinfection

Network #	Network Top Diseases and Functions	Score	#Focus Molecules
**1**	Organismal injury and abnormalities, Reproductive System Disease and Cancer	23	11
**2**	Organismal injury and abnormalities, Reproductive System Disease and Connective Tissue Disorders	20	10
**3**	Cell-To-Cell Signaling and Interaction, and Organismal injury and abnormalities	20	10
**4**	Cellular Function and Maintenance, Cell-To-Cell Signaling and Interaction, Inflammatory Response	18	9
**5**	Cancer, Organismal injury and abnormalities and Reproductive System Disease	15	8

Sequencing and Bioinformatics Information

An average Phred of 35 per base was used to assess sequencing quality, which is about one base error in about 2,500,000 reads.

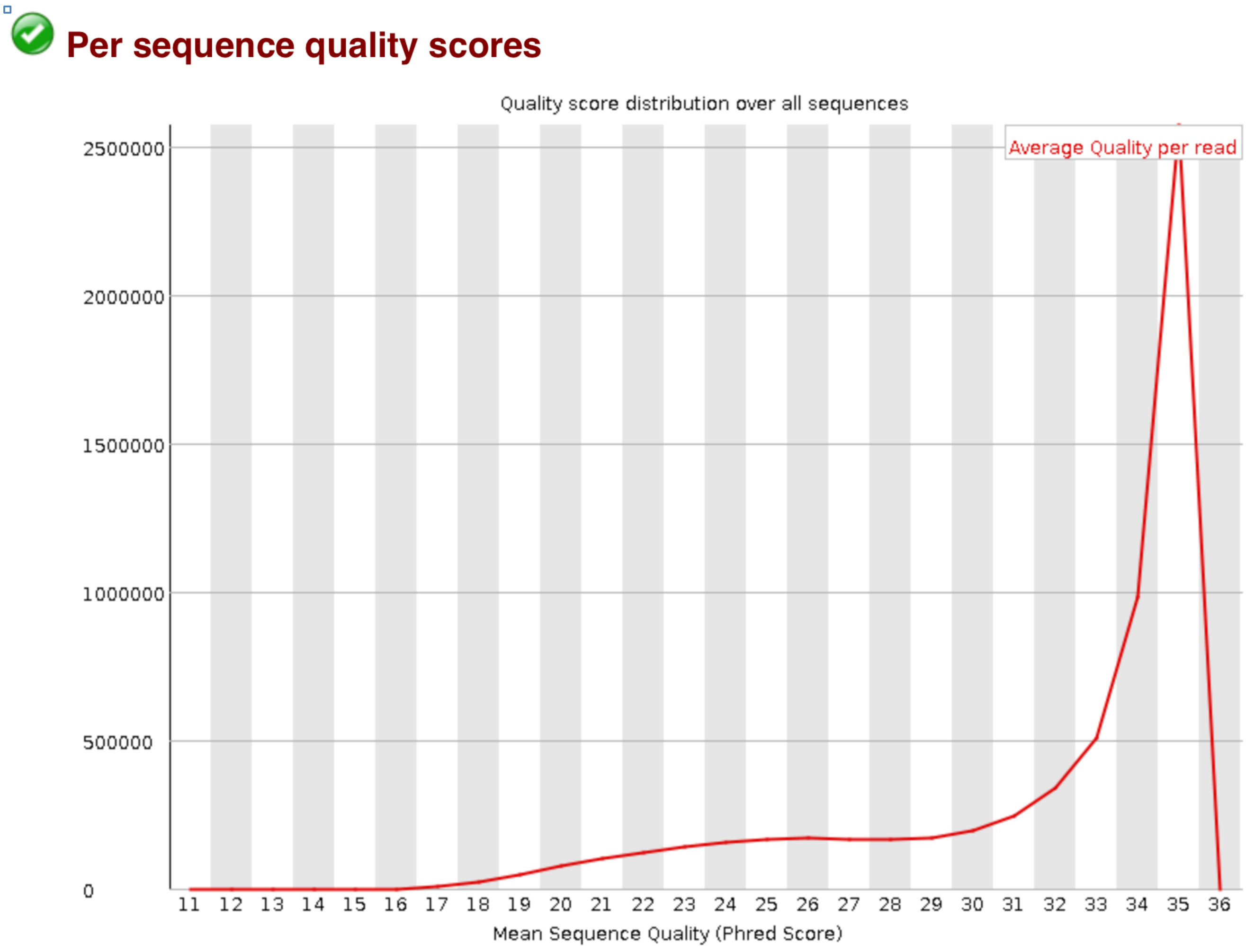

Figure S6: Example of quality scoring for miRNA sequencing in this study.

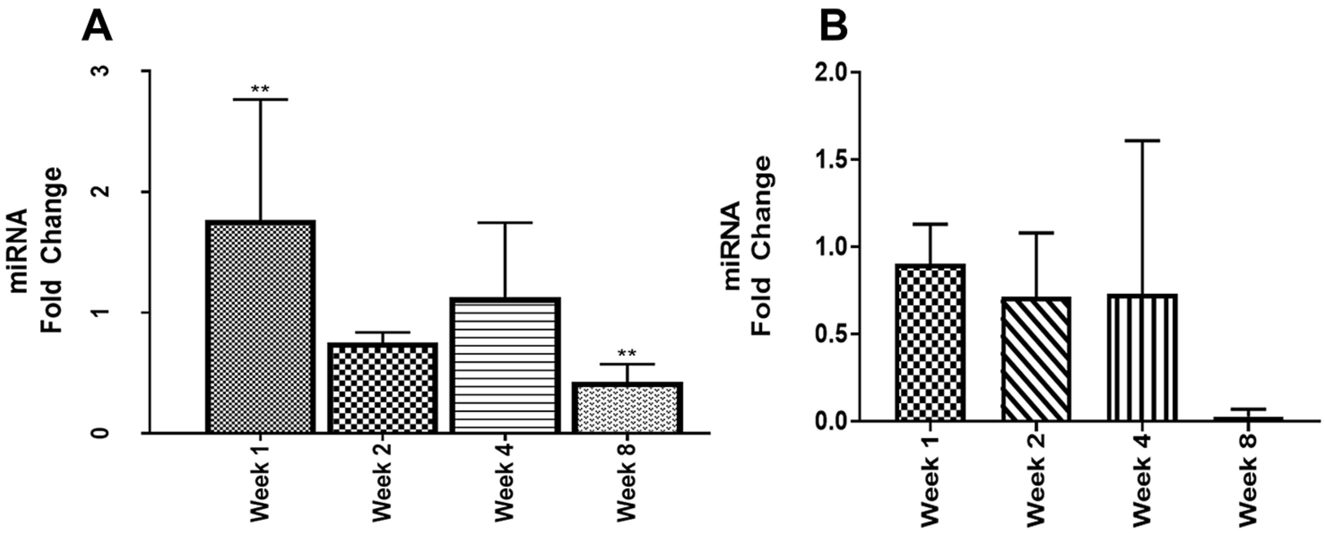

Figure S7: (A) Validation of miRNA 378b expression after single infection using qPCR, (B) Validation of miRNA 142-5p expression after single infection using qPCR.

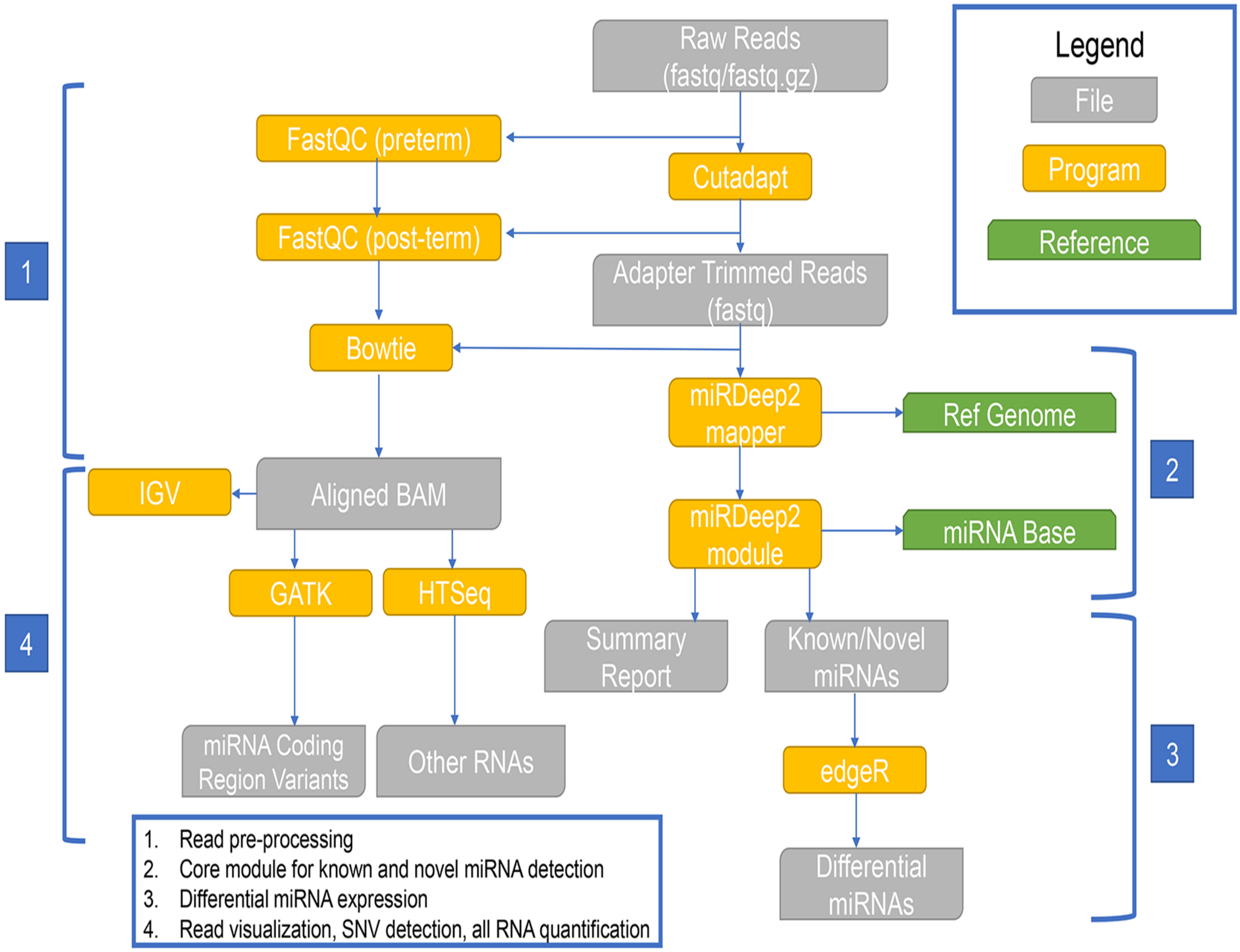

Figure S8: Comprehensive Analysis Pipeline for miRNA-seq data (CAP-miRSeq), adapted from Sun et al., 2014 (35).

Software tools

## Tool Paths

SCRIPT_PATH=/panfs/pstor.storage/rcclocal/zcluster/cap-mirseq/012014/scripts

MIRDEEP2_PATH=/panfs/pstor.storage/rcclocal/zcluster/cap-mirseq/012014/bin

BOWTIE_PATH=/panfs/pstor.storage/rcclocal/zcluster/cap-mirseq/012014/bin

RANDFOLD_PATH=/panfs/pstor.storage/rcclocal/zcluster/cap-mirseq/012014/bin

SQUID_PATH=/panfs/pstor.storage/rcclocal/zcluster/cap-mirseq/012014/bin

VIENNA_PATH=/panfs/pstor.storage/rcclocal/zcluster/cap-mirseq/012014/bin

PDFAPI2_PM_PATH=/panfs/pstor.storage/rcclocal/zcluster/cap-mirseq/012014/lib/perl5

JAVA_PATH=/usr/local/java/jdk6u3/jdk7/bin

NGS_PORTAL_PATH=/projects/bsi/bictools/apps/misc/ngs_dashboard/2.0

PICARD_PATH=/panfs/pstor.storage/rcclocal/zcluster/cap-mirseq/012014/bin

FASTQC_PATH=/panfs/pstor.storage/rcclocal/zcluster/cap-mirseq/012014/bin

CUTADAPT_PATH=/panfs/pstor.storage/rcclocal/zcluster/cap-mirseq/012014/bin

SAMTOOLS_PATH=/panfs/pstor.storage/rcclocal/zcluster/cap-mirseq/012014/bin

BEDTOOLS_PATH=/panfs/pstor.storage/rcclocal/zcluster/cap-mirseq/012014/bin

GATK_JAR=/usr/local/gatk/3.2.2/GenomeAnalysisTK.jar

VCFTOOLS_PATH=/panfs/pstor.storage/rcclocal/zcluster/cap-mirseq/012014/bin

VCFTOOLS_PERLLIB=/panfs/pstor.storage/rcclocal/zcluster/cap-mirseq/012014/lib/perl5/site_perl

HTSEQ_PATH=/panfs/pstor.storage/rcclocal/zcluster/cap-mirseq/012014/bin

HTSEQ_LIB_PATH=/usr/local/htseq/0.5.4p5/lib/python/HTSeq-0.5.4p5-py2.7-linux-x86_64.egg

PYTHON_PATH=/usr/local/python/2.7.2/bin

Tool Parameters

CUTADAPT_PARAMS=-b AATCTCGTATGCCGTCTTCTGCTTGC -b AGATCGGAAGAGCACACGTCTG -O 3 -m 17 -f fastq

MAPPER_PARAMS=-e -h -q -m -r 5 -u -v -o 4

MIRDEEP2_PARAMS=-P -t Mouse

MIRDEEP2_CLOSE_SPECIES=none

QUANTIFIER_PARAMS=-P -W

BOWTIE_PARAMS=-p 4 -S -q -n 1 -e 80 -l 30 -a -m 5 --best --strata

ADDORREPLACEREADGROUPS_PARAMS=MAX_RECORDS_IN_RAM=100000 VALIDATION_STRINGENCY=SILENT RGLB=mm10 RGCN=UGA RGPL=Illumina

SORTSAM_PARAMS=MAX_RECORDS_IN_RAM=1800000 VALIDATION_STRINGENCY=SILENT

PRINTREADS_PARAMS=-rf ReassignOneMappingQuality -RMQF 255 -RMQT 60

UNIFIEDGENOTYPER_PARAMS=-glm SNP -dcov 1000

HTSEQ_PARAMS=-m intersection-nonempty -q -t exon -s no

QUEUE=rcc-m128-30d

Reference Files

REF_GENOME=/home/qbcglab/qbcg/CAP_miR_files/mm10.fa

BOWTIE_REF=/home/qbcglab/qbcg/CAP_miR_files/mm10

MIRBASE_HAIRPIN=/home/qbcglab/qbcg/CAP_miR_files/hairpin.mmu.dna.fa

MIRBASE_MATURE=/home/qbcglab/qbcg/CAP_miR_files/mature.mmu.dna.fa

MIRBASE_GFF=/home/qbcglab/qbcg/CAP_miR_files/mmu.gff3

GENCODE_GTF=/home/qbcglab/qbcg/CAP_miR_files/gencode.vM2.annotation.gtf

## Memory Parameters

QSUB

REFERENCE_INDEXES_MEM=-l h_vmem=10G -l h_stack=10M

CUTADAPT_MEM=-l h_vmem=10G -l h_stack=10M

FASTQC_MEM=-l h_vmem=10G -l h_stack=10M

BAMS_MEM=-l h_vmem=10G -l h_stack=10M

MIRDEEP2_MAPPER_MEM=-l h_vmem=10G -l h_stack=10M

MIRDEEP2_MEM=-l h_vmem=10G -l h_stack=10M

VARIANTS_MEM=-l h_vmem=10G -l h_stack=10M

EXPRESSION_REPORTS_MEM=-l h_vmem=5G -l h_stack=10M

DIFF_EXPRESSION_MEM=-l h_vmem=10G -l h_stack=10M

GENCODE_CLASSIFICATION_MEM=-l h_vmem=20G -l h_stack=10M

SAMPLE_SUMMARY_MEM=-l h_vmem=10G -l h_stack=10M

MAIN_DOC_MEM=-l h_vmem=5G -l h_stack=10M

JVM

CREATEDICTIONARY_JVM_MEM=-Xmx5g -Xms1g

ADDORREPLACEREADGROUPS_JVM_MEM=-Xmx5g -Xms1g

SORTSAM_JVM_MEM=-Xmx5g -Xms1g

UNIFIEDGENOTYPER_JVM_MEM=-Xmx5g -Xms1g
